# Tools for Addressing Microaggressions: An Interactive Workshop for Perioperative Trainees

**DOI:** 10.15766/mep_2374-8265.11360

**Published:** 2023-11-28

**Authors:** LaMisha Hill Weller, Janette Tang, Rebecca Chen, Christy Boscardin, Odinakachukwu Ehie

**Affiliations:** 1 Associate Professor, Department of Obstetrics, Gynecology and Reproductive Sciences, University of California, San Francisco, School of Medicine; 2 Fourth-Year Medical Student, University of California, San Francisco, School of Medicine; 3 Professor, Departments of Medicine and Anesthesia and Perioperative Care, University of California, San Francisco, School of Medicine; 4 Associate Clinical Professor, Department of Anesthesia and Perioperative Care, University of California, San Francisco, School of Medicine

**Keywords:** Microaggression, Allyship, Bystander, Online/Distance Learning, Self-Assessment, Anti-racism, Diversity, Equity, Inclusion

## Abstract

**Introduction:**

Graduate trainees from diverse backgrounds may experience discrimination, mistreatment, and microaggressions. While the ability to identify and respond to microaggressions is a much-needed skill for all emerging trainees, limited training workshops exist for residents, especially within perioperative medicine. To embody the principles of diversity, equity, inclusion, and anti-racism (DEIA), we aimed to empower trainees in the perioperative environment with several strategies for addressing microaggressions to bridge this training gap.

**Methods:**

Based on critical race theory, transformative learning, minority stress theory, and the structural theory of gender and power, this workshop was developed with the primary aim of educating trainees on microaggressions, amplifying the role of allyship, and providing tools to respond to microaggressions as an ally. We used a mixed methods approach to examine participants’ pre/post self-evaluations of microaggression intervention tools and the overall effectiveness of the workshop.

**Results:**

The postsurvey captured the experiences of 54 trainees, including 37 of 44 (84%) first-year clinical anesthesia residents and 14 of 24 (58%) surgery residents. The facilitator and course feedback was remarkably positive. Paired *t* test analyses on participants’ pre- and postsurvey responses demonstrated a statistically significant increase in knowledge of microaggressions. This workshop also significantly increased learners’ self-reported tools for responding to microaggressions.

**Discussion:**

Overall, these promising findings suggest that the strategies presented in this workshop could be applied across other graduate medical education programs. Institutions may wish to customize workshop elements, such as the case scenarios, and the workshop can also be incorporated within a DEIA curriculum.

## Educational Objectives

By the end of this workshop, participants will be able to:

1. Describe three examples of microaggressions often experienced in the perioperative environment.
2. Identify eight strategies for addressing microaggressions.
3. Apply at least three strategies for addressing microaggressions during case scenario practices.
4. Practice one of the five direct strategies to address microaggressions in the perioperative environment during role-play in a small-group setting.
5. Demonstrate the skill of addressing microaggressions as an upstander in the perioperative environment.

## Introduction

The term *microaggressions* is used to describe subtle, biased statements, assumptions, and behaviors directed towards historically marginalized groups.^1^ It is widely known that graduate medical training can be stressful and that this can be compounded by the perioperative setting due to high-acuity patient care. These stressful dynamics can be exponential among perioperative trainees who are targets of microaggressions.^2,3^ A 2020 study examined racial and ethnic discrimination across 301 general surgery residency programs with a total of 6,956 active residents. Results found that 71% (171 of 242) of Black respondents, 46% (442 of 963) of Asian respondents, 33% (175 of 526) of other non-White respondents, and 25% (122 of 482) of Hispanic respondents reported experiencing discrimination during their time in residency. In this study, discriminatory behavior included being the target of slurs or hurtful comments (mostly from patients), being mistaken for nonphysicians (mostly by patients), being mistaken for another person of the same race (mostly by nurses/staff), and experiencing different standards of evaluation (mostly by attendees).^4^

Historically, the study of microaggressions has primarily focused on race and racism; however, a growing body of literature confirms that microaggressions also occur across gender, LGBTQIA+ communities, and other diverse social identities and cause harm in clinical and learning environments.^5,6^ Studies have linked microaggressions, identity-based harm, mistreatment, and discrimination with worse mental health outcomes,^5^ burnout,^2,7^ and decreased performance among diverse medical students during core clerkships.^8^ Furthermore, microaggressions have been associated with biased evaluations that could potentially result in long-term consequences for residency and career opportunities for diverse and historically underrepresented learners.^9^ Left unaddressed, these events could negatively impact and deteriorate institutional climate, inclusion, and belonging.

While many conceptually understand the definition of microaggressions, there is also a need to teach skills-based interventions for responding to microaggressions, especially for those who witness the microaggressions as a bystander but are not the target of the harm.^10–12^ To bridge this gap, we designed a microaggression workshop focusing on skill development as one module in a four-part diversity, equity, and inclusion (DEI) curriculum for perioperative residents at the University of California, San Francisco (UCSF).^13^ The curriculum design was informed by the theory of transformative learning, which is characterized by paradigm shifts that occur by “becoming aware of prior assumptions and having one's world view challenged.”^14^ This results in changed beliefs about oneself, others, and other social schemas.^15,16^ The curriculum was also grounded in critical race theory (CRT), a multidimensional framework originating in legal studies and social justice. CRT's primary objective is to examine the relationship among race, racism, and power. CRT embodies anti-racism while attending to intersectionality and the compounded systems of oppression.^17^ In addition, minority stress theory highlights barriers, discrimination, and disparities experienced by people who identify as gender and sexual minorities.^18^ Finally, the structural theory of gender and power addresses imbalances between men and women across dimensions of gender relations.^19^ Combined, these frameworks center on raising awareness of other people's lived experiences, particularly those from historically marginalized backgrounds.

We identified six relevant publications in *MedEdPORTAL* focusing on microaggression or mistreatment training for GME trainees.^20–25^ None of these were explicitly designed for residents in the perioperative environment. Furthermore, the frameworks presented were primarily designed to guide participants through an internal process and relied upon common communication skills. Our workshop furthers the existing body of literature on microaggressions in GME by presenting a curated list of best-practice tools to address microaggressions across a skills-based continuum. We have selected three indirect strategies (redirect, uplift, and besting) as lower-risk tools to disrupt microaggressions. As perioperative residents progress through their training and acquire more power and privilege within their professional status, they require tools to engage in diversity dialogues and address microaggressions when they occur. Therefore, we also provide five direct strategies (reflect back, communicate impact, raise awareness, check-in, and reaffirm boundaries) that invite dialogue and actively address microaggressions. We also emphasize the need to shift the burden of responding to microaggressions away from the individual who has been harmed. Our training raises awareness of the importance of allyship and encourages perioperative residents who witness microaggressions to take action.

## Methods

Based on Kern's six-step approach to curriculum development in medical education, the methodological framework that we used to teach perioperative trainees about microaggression is presented below.^26^

### Step 1: Needs Assessment

We examined the current literature on discrimination, mistreatment, and microaggressions in graduate medical education.^2–4,20–25^ We identified a need to curate a training for perioperative residents that would offer best-practice tools to address microaggressions across a skills-based continuum.

### Step 2: Targeted Needs Assessment

We combined a targeted needs assessment survey with a presurvey to minimize residents receiving multiple surveys and increase the response rate. For the targeted needs assessment survey, we inquired about what an ideal curriculum would look like. The presurvey examined the learning objectives before we administered the workshop. We administered the combined needs assessment and presurvey (Appendix A) immediately following a 1-hour didactic introductory session for first-year clinical anesthesia (CA 1) residents in 2020 and 2021. Due to scheduling conflicts, we also sent this survey via email to CA 2, CA 3, and surgery residents during their research year 4–6 weeks prior to the start of the 2020 curriculum to capture as much information as we could around the targeted needs assessment. The introductory session provided a brief didactic on national data on discrimination and mistreatment and reviewed the upcoming four-part workshop series goals and objectives.^13^ In total, 83% (79 of 95) of 2020 participants (CA 1, CA 2, CA 3, and surgical residents) and 68% (17 of 25) of 2021 participants (new CA 1 residents) completed the needs assessment and presurvey (Appendix A). Not only did most participants report they had not previously received formal DEI training, they also identified that actionable changes, tools, interactive activities to uncover biases, and small-group discussions were important. We also created a postsurvey (Appendix B), a self-reflective exercise (Appendix C), a learner's guide (Appendix D), a facilitator guide (Appendix E), a list of tools for addressing microaggressions (Appendix F), and didactic slides (Appendix G) for the workshop.

### Step 3: Goals and Objectives

During the 2-hour workshop, participants were presented with current literature on microaggressions via the didactic PowerPoint (Appendix G). The experiential sections of the workshop featured tools for responding to microaggressions, relevant clinical case scenarios, and self-reflective small-group exercises (Educational Objectives 1–4; Appendix G). The workshop's aim was to provide evidence-based foundations on the negative impact of microaggressions. In addition, we prioritized facilitated small-group spaces for providers to discuss their lived experiences surrounding microaggressions, uplift the role of allies in addressing microaggressions, and practice tools to disrupt such events (Educational Objective 5). We wanted participants to gain experience with discussing microaggressions and other diversity themes as a standard part of medical education. Most importantly, we emphasized the need for all professional members to act as allies and speak up when they observed microaggressions.

### Step 4: Educational Strategies

#### Didactic and experiential activities

The core workshop elements were designed to transform didactic knowledge into applied behaviors. One to 2 weeks prior to the workshop, we emailed the residents a learner's guide that contained required prereadings (Appendix D).^25,27,28^ We provided facilitators with a detailed teaching guide (Appendix E) to review prior to the session, slides (Appendix G) to use for the didactic portion of the workshop, and a tool guide (Appendix F) to screen-share within the breakout groups as residents practiced skills in addressing microaggressions in relevant case scenarios.

As seen in Appendix G, the workshop included community agreements, brief didactic information with relevant research, clinically pertinent case scenarios, computer-based poll questions, and facilitated small-group discussions. To further crystallize skills, the reflective exercise in Appendix C was utilized. As a strategy to foster audience engagement, we wove the interactive components throughout the workshop. Due to the COVID-19 pandemic, we conducted this workshop virtually via Zoom, which allowed the lead facilitators to leverage the poll feature as well as other technological advantages. Using the Zoom poll feature, we shared the breakdown of workshop participants’ responses to two poll questions to reflect the different perspectives on which tool each participant would use to address the microaggression in case scenario 1 (Appendix G, slides 11–12).

#### Case scenarios

We gathered clinically relevant case scenarios and deidentified them for privacy. Using questions from the facilitator guide in Appendix E, the case scenarios explored themes of gender, race, ethnicity, LGBTQIA+, and other intersectionalities. Participants role-played potential responses and discussed issues of identity, power, and privilege.

#### Microaggression intervention tools

We assembled a list of microaggression tools from evidence-based best practices and frameworks surrounding microaggressions, interventions, bystander-upstander, and communication skills.^11,12,27,28^ The interventions outlined in Appendix F included a list of indirect and direct strategies to address microaggressions set along a skills-based continuum.

#### Experiential facilitated small groups

The experiential small-group discussions were facilitated by faculty members representing diverse lived experiences across gender, race, ethnicity, religion, and LGBTQIA+ identities. Faculty facilitators invited participants to share their reactions to each case scenario. Participants reflected on the impact of not addressing microaggressions. Participants also identified the tools they would use to respond to the actor of the microaggression and practiced being an upstander in a role-play with other participants in their small group.

### Step 5: Implementation

#### Recommended preworkshop resources

During the weeks prior to the session, participants received a learner's guide to reference along with workshop objectives and other relevant information (Appendix D).

#### Facilitator selection and training

Faculty members across six different disciplines who had previously demonstrated commitments to engaging in DEI initiatives served as small-group workshop facilitators. Faculty facilitators participated in a 1-hour, preparatory, train-the-trainer session to review the workshop content in Appendix E and familiarize themselves with the experiential exercises and small-group activities. Facilitators discussed ways to manage challenging themes that could arise during the workshop.

#### Workshop administration

This workshop was administered during protected educational nonclinical time embedded within the residency program known as Anesthesia Education Day. This recurrent educational block occurred bimonthly in two groups, with one-half of the class attending in alternating weeks. Due to the pandemic and scheduling restrictions, the workshop was only available to first-year anesthesia residents concurrently with orthopedic surgery and general surgery residents in their research year.

### Step 6: Evaluation

#### Evaluation design and participants

We utilized a pre/post design to explore the efficacy of the workshop. The core evaluation components were modeled after the first two levels of the Kirkpatrick framework,^29^ satisfaction (level 1) and competency (level 2). Combined, the pre/post design and the workshop didactic and experiential activities aimed to assess the participants’ level of satisfaction, knowledge gained, and self-assessment of confidence in using applied behavioral tools for addressing microaggressions. This educational activity was designated as exempt by the UCSF Institutional Review Board (approval #19-29554, May 14, 2020).

Prior to administration, the survey items were reviewed by two anesthesia fellows to assess their overall clarity, revised, and retested on a sample of 10 second-year and six fourth-year medical students, followed by a 30-minute interview with each student to assess for validity of each question item. The combined targeted needs assessment survey and presurvey (Appendix A) were administered 4–6 weeks before the workshop as a method to establish a baseline for the knowledge domains of the full curriculum. The postsurvey (Appendix B) was distributed to participants as the final activity of the workshop and included parallel questions surrounding overall workshop satisfaction (level 1), knowledge, and self-assessment of tools to address microaggressions (level 2).

#### Data analysis

All responses to surveys and reflection exercises were collected using Qualtrics software. Data gathered via pre/post assessments were examined using a paired *t* test analysis. The pre- and postsurvey responses were matched with anonymous identification. In addition, open-ended participant feedback on the most effective aspects of the workshop and areas for improvement was summarized for themes.

## Results

### Participant Demographics

This workshop was administered to 44 out of 50 anesthesia CA 1 and 24 out of 30 surgery PGY 4/5 resident trainees over the course of four separate sessions in November 2020 and November 2021. The postsurvey captured the experiences of 54 trainees, including 37 of 44 (84%) anesthesia CA 1 and 14 of 24 (58%) surgery residents. Among anesthesia respondents, 19 (51%) identified as female, seven (19%) identified as LGBTQIA+, and 15 (40%) identified as underrepresented in medicine (UIM). Among surgery respondents, seven (50%) identified as female, two (14%) identified as LGBTQIA+, and five (36%) identified as UIM (Table 1).

**Table 1.**
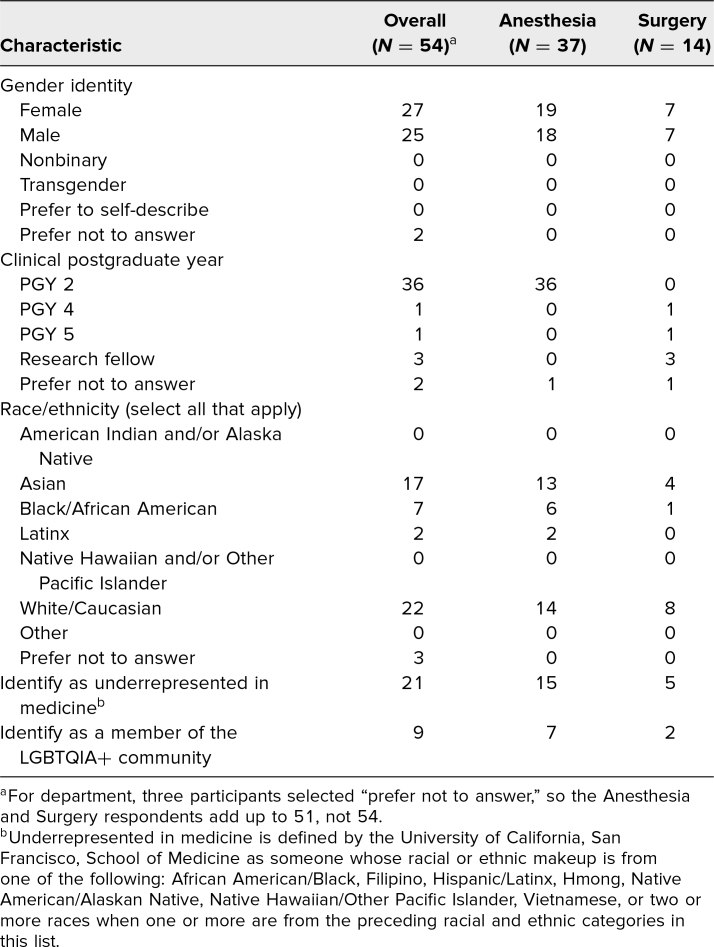
Demographics of Workshop Participants

### Workshop and Facilitator Satisfaction

Responses regarding satisfaction with this workshop and its facilitators were overwhelmingly positive in the postsurvey. The statements, graded on a 5-point Likert scale (1 = *strongly disagree,* 5 = *strongly agree*), revealed a mean of 4.6 (*SD* = 0.9) for “This microaggression workshop is important to my training,” 4.8 (*SD* = 0.7) for “I believe this microaggression workshop is relevant to my workplace,” and 4.8 (*SD* = 0.6) for “I would recommend this microaggression workshop to my peers.” Statements for facilitator feedback resulted in a mean of 4.9 (*SD* = 0.3) for “The facilitators were well prepared,” 4.9 (*SD* = 0.4) for “The facilitators created a welcoming and inclusive environment for discussions,” and 5.0 (*SD* = 0.0) for “The facilitators effectively communicated this information” (Table 2).

**Table 2.**
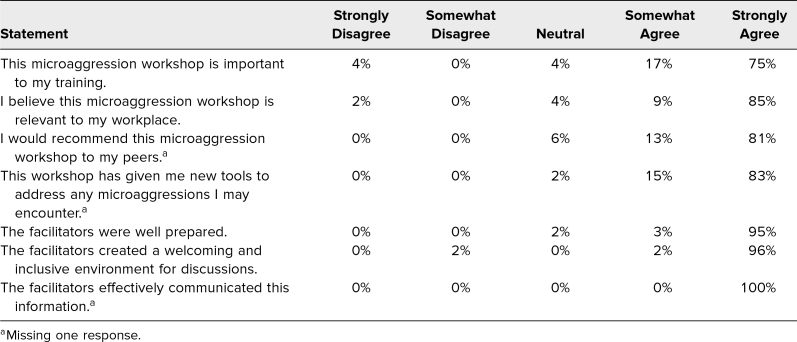
Workshop and Facilitator Feedback (*N* = 54)

### Self-Perceived Assessment of Tools for Disrupting Microaggressions

We assessed self-perceived competence through responses to four statements on the postsurvey rated on a 5-point Likert scale (1 = *strongly disagree,* 5 = *strongly agree*). The statement “I am likely to take action at the time I witness a microaggression addressed towards others” increased from a mean of 2.6 (*SD* = 1.1) to 4.3 (*SD* = 0.6; *p* < .05) from pre- to postsurvey, respectively. The statement “I feel that I have the tools to address the microaggressions I witness” increased from a mean of 2.7 (*SD* = 1.1) to 4.6 (*SD* = 0.5; *p* < .05). The statement “I am likely to take action at the time I receive a microaggression” increased from a mean of 2.2 (*SD* = 1.0) to 4.2 (*SD* = 0.8; *p* < .05). The statement “I feel that I have the tools to address the microaggression at the time I receive it” increased from a mean of 2.6 (*SD* = 1.1) to 4.5 (*SD* = 0.6; *p* < .05; Figure).

**Figure.**
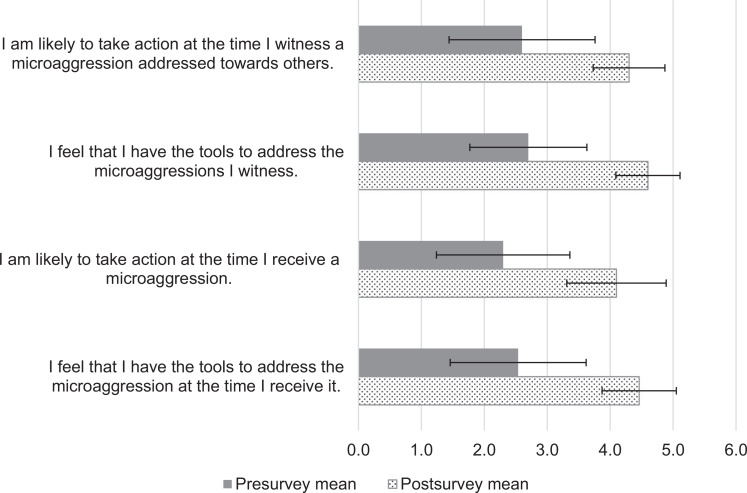
Mean pre-/postworkshop change in self-perceived competence (*N* = 55). Rated on a 5-point Likert scale (1 = *strongly disagree,* 5 = *strongly agree*). Error bars indicate ±1 standard deviation.

### Open-Ended Workshop Feedback and Self-Reflection

At the end of the postsurvey, participants were presented with three open-ended questions to capture additional workshop feedback and self-reflections in a free-response format. Forty-four of 54 participants submitted responses to the question “What did you like most about this workshop?” The most prominent themes in their responses, by number of appearances, included the following: learning about practical tools and skills (50% of responses), role-playing scenarios (30%), and small-group discussions (25%). The 28 responses to the question “What could be improved in this workshop?” can be summarized by the following themes: nothing (32%), more resources for responding to different types of microaggressions (32%), more cases/tools for addressing power differentials (14%), and more small-group discussions (14%). Eighteen participants submitted self-reflections on the question “What did you learn about yourself?” Their reflections included the following themes: I am uncomfortable with confrontation (50%), and I have a lot to learn about being an ally and how to respond to microaggressions (39%; Table 3).

**Table 3.**
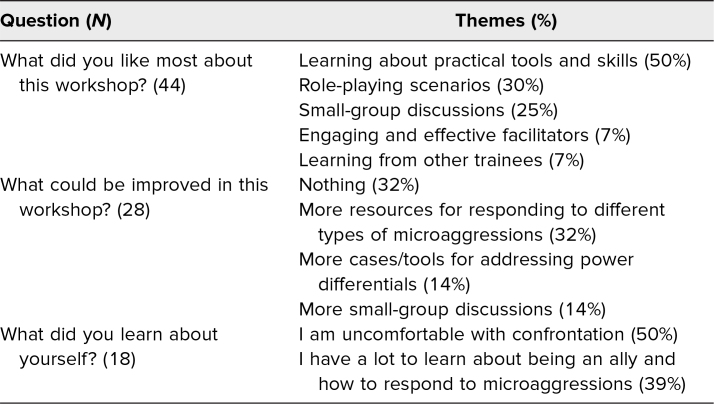
Themes Derived From Workshop Feedback

## Discussion

This microaggression workshop provided an important contribution to graduate medical education, offered intervention tools to support upstander diversity skills, and was curated specifically for perioperative residents. Results indicate that the facilitators were prepared, were effective in content delivery, and created an intimate space for discussions. We utilized a ratio of 1:5 facilitators to learners to allow for breakout small-group dialogues. In addition, the train-the-trainer session prior to the workshop supported facilitator preparedness, demonstrated faculty commitment to diversity, and increased each department's internal capacity to engage in diversity educational efforts. The data also suggest that participants furthered their ability to define microaggressions, gained additional tools to address microaggressions they might experience directly or witness, and would be more likely to take action to address microaggressions they might experience directly or witness.

Our workshop was grounded in didactic, evidence-based content on microaggressions and incorporated clinically relevant case scenarios from the perioperative environment. We also selected best-practice tools for responding to microaggressions set along a skills-based continuum to support the professional development of trainees. Finally, our workshop utilized experiential self-reflective exercises, and participants were very engaged in the small-group discussions.

There were also limitations to the workshop. First, the evaluation relied on self-reported assessment and did not include an independent rater or formal evaluation of skills in responding to microaggressions. Second, we were unable to assess change over time and determine if the workshop altered resident behavior in responding to microaggressions in the perioperative environment. Further research would benefit from longer-term follow-up to assess both knowledge retention and behavioral change in applying the microaggression tools. In addition, the open-ended questions demonstrated that participants preferred more time in small groups and wanted additional resources for tools to address microaggressions. Future workshops would benefit from allocating more time to the small-group breakout sessions, where the vulnerable conversations occur.

Adopters of this workshop are encouraged to engage additional facilitators to allow the primary workshop leaders to effectively deliver the session content and manage any needed in-session logistics (poll questions, virtual breakout rooms, handouts, etc.). In addition, maintaining a group size of four to six participants per facilitator supports the ability of everyone to share and engage. One should also consider racial affinity groups (i.e., UIM vs. non-UIM) if possible. During the workshop, it is recommended that more time be allotted for small groups and that the small groups remain intact (rather than being continuously randomized per activity) to support relationship building, comfort, and group cohesion. Finally, we encourage those who adopt this workshop to also explore ways to gather clinically and institutionally relevant case scenarios with permission in order to increase awareness and support engagement in the workshop.

Microaggression is a broad framework that can be experienced differently across social identities and systems rooted in hierarchy such as academic medicine. However, as perioperative trainees mature, they have an obligation to develop the skills to address microaggressions as our next generation of faculty leaders.

## Appendices


Needs Assessment and Presurvey.docxPostsurvey.docxReflective Exercise.docxLearners Guide.docxFacilitator Guide.docxTools to Address Microaggression.pdfMicroaggression Workshop Presentation.pptx
*All appendices are peer reviewed as integral parts of the Original Publication.*
