# Supplementary material for: Tools for Addressing Microaggressions: An Interactive Workshop for Perioperative Trainees
Source: MedEdPORTAL. 2023 Nov 28;19:11360. doi: 10.15766/mep_2374-8265.11360 (PMC10682127; doi:10.15766/mep_2374-8265.11360)
Supplement: Supplementary file 1 — Needs Assessment and Presurvey.docxPostsurvey.docxReflective Exercise.docxLearners Guide.docxFacilitator Guide.docxTools to Address Microaggression.pdfMicroaggression Workshop Presentation.pptx [file mep_2374-8265.11360-s001.zip › A. Needs Assessment and Presurvey.docx]

**DEI Needs Assessment**

**Start of Block: Default Question Block**

1 Create and enter an anonymous ID:
1. First 3 letters of your current address
2. Last 2 letters of birth city
3. Last digit of year of birth
4. Number of siblings (not including self)
Example: Red, San Francisco, 1991, 3 brothers = REDCO13

________________________________________________________________

2 **If you were NOT present at the introductory session, please skip this question.**
 
The following statements assess your satisfaction towards the introduction session. How strongly do you agree with the following?

|  | Strongly disagree (1) | Somewhat disagree (2) | Neutral (3) | Somewhat agree (4) | Strongly agree (5) |
| --- | --- | --- | --- | --- | --- |
| This introductory workshop showed me that a DEI curriculum is important to my training. (1) |  |  |  |  |  |
| I believe this workshop is relevant to my workplace. (2) |  |  |  |  |  |
| I would recommend this workshop to my peers. (3) |  |  |  |  |  |

3 The following questions are designed to explore your personal experiences related to microaggressions in the workplace.

4 Have you ever **experienced** a microaggression?

- Yes (1)
- No (2)
- I don't know or I'm not sure what a microaggression is. (3)

*Display This Question:*

*If Have you ever experienced a microaggression? = Yes*

5 The following questions are designed to explore your experiences **receiving** microaggressions (e.g., between colleagues, patients, etc.) in the workplace, and NOT witnessing microaggressions.

*Display This Question:*

*If Have you ever experienced a microaggression? = Yes*

6 Where have you **experienced** microaggressions?

- At your local institution (1)
- Outside of your local institution (2)
- Both (3)

*Display This Question:*

*If Have you ever experienced a microaggression? = Yes*

7 In the past 30 days, how often have you **experienced** microaggressions at work?

- 0 (1)
- 1-2 (2)
- 3-4 (3)
- 5-6 (4)
- 7+ (5)

*Display This Question:*

*If Have you ever experienced a microaggression? = Yes*

8 What aspects of your identity do these microaggressions involve?

- Race/ethnicity (1)
- Gender (2)
- Sexual Orientation (3)
- Other: (4) ________________________________________________

9 How strongly do you agree with the following?

|  | Strongly Disagree (1) | Somewhat Disagree (2) | Neutral (3) | Somewhat Agree (4) | Strongly Agree (5) |
| --- | --- | --- | --- | --- | --- |
| I know how to define the term *microaggression*. (1) |  |  |  |  |  |
| I feel that I have the tools to address the *microaggression* at the time I receive it. (2) |  |  |  |  |  |
| I am likely to take action at the time I receive a *microaggression*. (3) |  |  |  |  |  |

10 Have you ever **witnessed** a microaggression?

- Yes (1)
- No (2)
- I don't know or I'm not sure what a microaggression is. (3)

*Display This Question:*

*If Have you ever witnessed a microaggression? = Yes*

11 The following questions are designed to explore your experiences **witnessing** microaggressions (e.g., between colleagues, patients, etc.) in the workplace, and NOT receiving microaggressions.

*Display This Question:*

*If Have you ever witnessed a microaggression? = Yes*

12 Where have you **witnessed** microaggressions?

- At your local institution (1)
- Outside of your local institution (2)
- Both (3)

*Display This Question:*

*If Have you ever witnessed a microaggression? = Yes*

13 In the past 30 days, how often have you **witnessed** microaggressions at work?

- 0 (1)
- 1-2 (2)
- 3-4 (3)
- 5-6 (4)
- 7+ (5)

14 How strongly do you agree with the following?

|  | Strongly Disagree (1) | Somewhat Disagree (2) | Neutral (3) | Somewhat Agree (4) | Strongly Agree (5) |
| --- | --- | --- | --- | --- | --- |
| I feel that I have the tools to address the *microaggressions* I witness. (1) |  |  |  |  |  |
| I am likely to take action at the time I witness a *microaggression* addressed towards others. (2) |  |  |  |  |  |

15 How strongly do you agree with the following?

|  | Strongly Disagree (1) | Somewhat Disagree (2) | Neutral (3) | Somewhat Agree (4) | Strongly Agree (5) |
| --- | --- | --- | --- | --- | --- |
| I know how to define the term *unconscious bias.* (1) |  |  |  |  |  |
| I have *unconscious biases.* (2) |  |  |  |  |  |
| My *unconscious biases* affect my clinical practice and/or interactions with others. (3) |  |  |  |  |  |
| I actively reflect on my *unconscious biases.* (8) |  |  |  |  |  |
| My self-awareness of my *unconscious biases* motivates me to interact with peers from different social backgrounds in the workplace. (4) |  |  |  |  |  |

16 How strongly do you agree with the following?

|  | Strongly Disagree (1) | Somewhat Disagree (2) | Neutral (3) | Somewhat Agree (4) | Strongly Agree (5) |
| --- | --- | --- | --- | --- | --- |
| I know how to define the term *allyship*. (1) |  |  |  |  |  |
| As an ally, I am likely to mentor individuals that belong to a marginalized group and recommend them for other academic projects. (2) |  |  |  |  |  |
| I recognize the privilege that I have. (4) |  |  |  |  |  |

17 The following True/False questions are meant to evaluate your general understanding of the terms, and not what you would do personally.

18 Unconscious bias is defined as a strong inclination of the mind or a preconceived opinion about someone or something.

- True (1)
- False (2)
- I'm not sure (3)

19 Stereotype suppression is the active suppression of stereotypical thoughts about an individual of a different social background that usually results in increased social encounters.

- True (1)
- False (2)
- I'm not sure (3)

20 Performative allyship is when someone from a non-marginalized group professes solidarity with a marginalized group in a way that is not helpful to that marginalized group.

- True (1)
- False (2)
- I'm not sure (3)

21 Microaggression is the brief subtle snub of any individual that is usually intentional and has negative impact.

- True (1)
- False (2)
- I'm not sure (3)

22 My training up to this point has prepared me to address conflicts regarding:

|  | Strongly disagree (1) | Somewhat disagree (2) | Neutral (3) | Somewhat agree (4) | Strongly agree (5) |
| --- | --- | --- | --- | --- | --- |
| Race/ethnicity (1) |  |  |  |  |  |
| Gender (2) |  |  |  |  |  |
| Sexual orientation (3) |  |  |  |  |  |

23 In the past year, how frequently did you confront microaggressions expressed by patients regarding:

|  | Never (1) | A few times a year (2) | A few times a month (3) | A few times a week (4) | Every day (5) |
| --- | --- | --- | --- | --- | --- |
| Race/ethnicity (1) |  |  |  |  |  |
| Gender (2) |  |  |  |  |  |
| Sexual orientation (3) |  |  |  |  |  |

24 In the past year, how frequently did you confront microaggressions expressed by residents regarding:

|  | Never (1) | A few times a year (2) | A few times a month (3) | A few times a week (4) | Every day (5) |
| --- | --- | --- | --- | --- | --- |
| Race/ethnicity (1) |  |  |  |  |  |
| Gender (2) |  |  |  |  |  |
| Sexual orientation (3) |  |  |  |  |  |

25 In the past year, how frequently did you confront microaggressions expressed by attendings regarding:

|  | Never (1) | A few times a year (2) | A few times a month (3) | A few times a week (4) | Every day (5) |
| --- | --- | --- | --- | --- | --- |
| Race/ethnicity (1) |  |  |  |  |  |
| Gender (2) |  |  |  |  |  |
| Sexual orientation (3) |  |  |  |  |  |

26 Please answer the following 4 questions in the context of life in general, and not just in work settings.

27 Choose the response that is most applicable to you up to this point:

- I **talk** to others who look like me. (1)
- I **listen** to others who look differently than me. (2)
- I **socialize** with others who look differently than me. (3)

28 Choose the response that is most applicable to you up to this point:

- I **talk** to others who think like me. (1)
- I **listen** to others who think differently than me. (2)
- I **socialize** with others who think differently than me. (3)

29 Choose the response that is most applicable to you up to this point:

- I strive to be comfortable and don't usually address my own biases. (1)
- I understand my own biases and knowledge gaps and share them with others. (2)
- I don't let mistakes from my own biases deter me from continuing to critically evaluate my own biases. (3)

30 Choose the response that is most applicable to you at this point:

- I **avoid** hard questions about privilege and racism. (1)
- I **understand** my own privilege in ignoring racism. (2)
- I **speak out** when I see racism in action. (3)

31 Have you received formal Diversity, Equity, and Inclusion (DEI) training before?

- Yes (1)
- No (2)

*Display This Question:*

*If Have you received formal Diversity, Equity, and Inclusion (DEI) training before? = Yes*

32 What was most effective in that DEI training?

________________________________________________________________

33 What does an ideal DEI training look like for you?

________________________________________________________________

34 Anything else you'd like to share?

________________________________________________________________

**End of Block: Default Question Block**

**Start of Block: Demographics**

35 Options for identities are abbreviated to preserve survey-takers' anonymity.

36 What department are you in?

- Anesthesia (1)
- Surgery (2)
- Prefer Not to Answer (3)

*Display This Question:*

*If What department are you in? = Anesthesia*

37 Which year of anesthesia postgraduate training are you?

- Intern (1)
- CA-1 (2)
- CA-2 (3)
- CA-3 (4)
- Fellow (5)
- Prefer not to answer (6)

*Display This Question:*

*If What department are you in? = Surgery*

38 Which year of surgery postgraduate training are you in?

- PGY-1 (1)
- PGY-2 (2)
- PGY-3 (3)
- Research Fellow (4)
- PGY-4 (5)
- PGY-5 (6)
- Prefer Not to Answer (7)

39 Race/ethnicity: (Choose all that apply)

- White/Caucasian (1)
- Black/African American (2)
- American Indian and/or Alaska Native (3)
- Asian (4)
- Native Hawaiian and/or other Pacific Islander (5)
- Latinx (6)
- Other (7) ________________________________________________
- Prefer not to answer (8)

40 Do you identify as Underrepresented In Medicine as defined by your School of Medicine? 

 *Includes: African American/Black, Asian (Filipino, Hmong, or Vietnamese only), Hispanic/Latinx, Native American/Alaskan Native, Native Hawaiian/Other Pacific Islander, or two or more races (when one or more are from the preceding racial and ethnic categories in this list)*

- Yes (1)
- No (2)
- Prefer not to answer (3)

41 Gender Identity: Choose all that apply.

- Male (1)
- Female (2)
- Non-binary (3)
- Transgender (4)
- Prefer to Self-Describe (5) ________________________________________________
- Prefer Not to Answer (6)

42 Do you identify as a member of the LGBTQIA+ Community?

- Yes (1)
- No (2)
- Prefer not to answer (3)

**End of Block: Demographics**

Adapted from validated study:

Hu Y-Y, Ellis RJ, Hewitt DB, et al. Discrimination, Abuse, Harassment, and Burnout in Surgical Residency Training. *N Engl J Med*. 2019;381(18):1741-1752. doi:10.1056/NEJMsa1903759.
