# Supplementary material for: Tools for Addressing Microaggressions: An Interactive Workshop for Perioperative Trainees
Source: MedEdPORTAL. 2023 Nov 28;19:11360. doi: 10.15766/mep_2374-8265.11360 (PMC10682127; doi:10.15766/mep_2374-8265.11360)
Supplement: Supplementary file 1 — Needs Assessment and Presurvey.docxPostsurvey.docxReflective Exercise.docxLearners Guide.docxFacilitator Guide.docxTools to Address Microaggression.pdfMicroaggression Workshop Presentation.pptx [file mep_2374-8265.11360-s001.zip › C. Reflective Exercise.docx]

**Reflective Exercise – Microaggression**

What tools did you feel most comfortable using in addressing the microaggression in the second case scenario after practicing in your small group setting?
