# Supplementary material for: Tools for Addressing Microaggressions: An Interactive Workshop for Perioperative Trainees
Source: MedEdPORTAL. 2023 Nov 28;19:11360. doi: 10.15766/mep_2374-8265.11360 (PMC10682127; doi:10.15766/mep_2374-8265.11360)
Supplement: Supplementary file 1 — Needs Assessment and Presurvey.docxPostsurvey.docxReflective Exercise.docxLearners Guide.docxFacilitator Guide.docxTools to Address Microaggression.pdfMicroaggression Workshop Presentation.pptx [file mep_2374-8265.11360-s001.zip › F. Tools to Address Microaggression.pdf]

Table 2. Examples of indirect and direct strategies to address microaggressions

**Indirect Strategies**	**Direct Strategies**
Redirect<br>Change the subject or shift attention away from the target.	Reflect Back<br>*'I heard you say ___, can you explain what you mean by ____.'*
Uplift<br>Elevate the target by countering the microaggression with affirmation, *'You are partnered with an amazing provider and are so fortunate to have them taking care of you.'*	Communicate Impact<br>*'Using words like ___ makes me feel uncomfortable.'*
Besting [39]<br>Use affirming counter-narratives to oppose microaggressions. *'Historically Black Colleges and Universities (HBCUs) matriculate the highest percentage of Black doctors.'*	Raise Awareness<br>*'I believe a more inclusive term is ___.'*
	Check-In (with the person who committed a microaggression):<br>*'I've been reflecting on the conversation from our team meeting yesterday. Can we find some time to check-in?'*
	Reaffirm Boundaries<br>*'We do not tolerate that kind of language, and I ask that everyone speak with respect.'*

Ehie O, Muse I, Hill L, Bastien A. Professionalism: microaggression in the healthcare setting. *Curr Opin Anesthesiol*. 2021;34(2):131-136. doi:10.1097/ACO.0000000000000966

*This is from an open access/public domain journal article.
