# Supplementary material for: Tools for Addressing Microaggressions: An Interactive Workshop for Perioperative Trainees
Source: MedEdPORTAL. 2023 Nov 28;19:11360. doi: 10.15766/mep_2374-8265.11360 (PMC10682127; doi:10.15766/mep_2374-8265.11360)
Supplement: Supplementary file 1 — Needs Assessment and Presurvey.docxPostsurvey.docxReflective Exercise.docxLearners Guide.docxFacilitator Guide.docxTools to Address Microaggression.pdfMicroaggression Workshop Presentation.pptx [file mep_2374-8265.11360-s001.zip › G. Microaggression Workshop Presentation.pptx]

## Slide 1
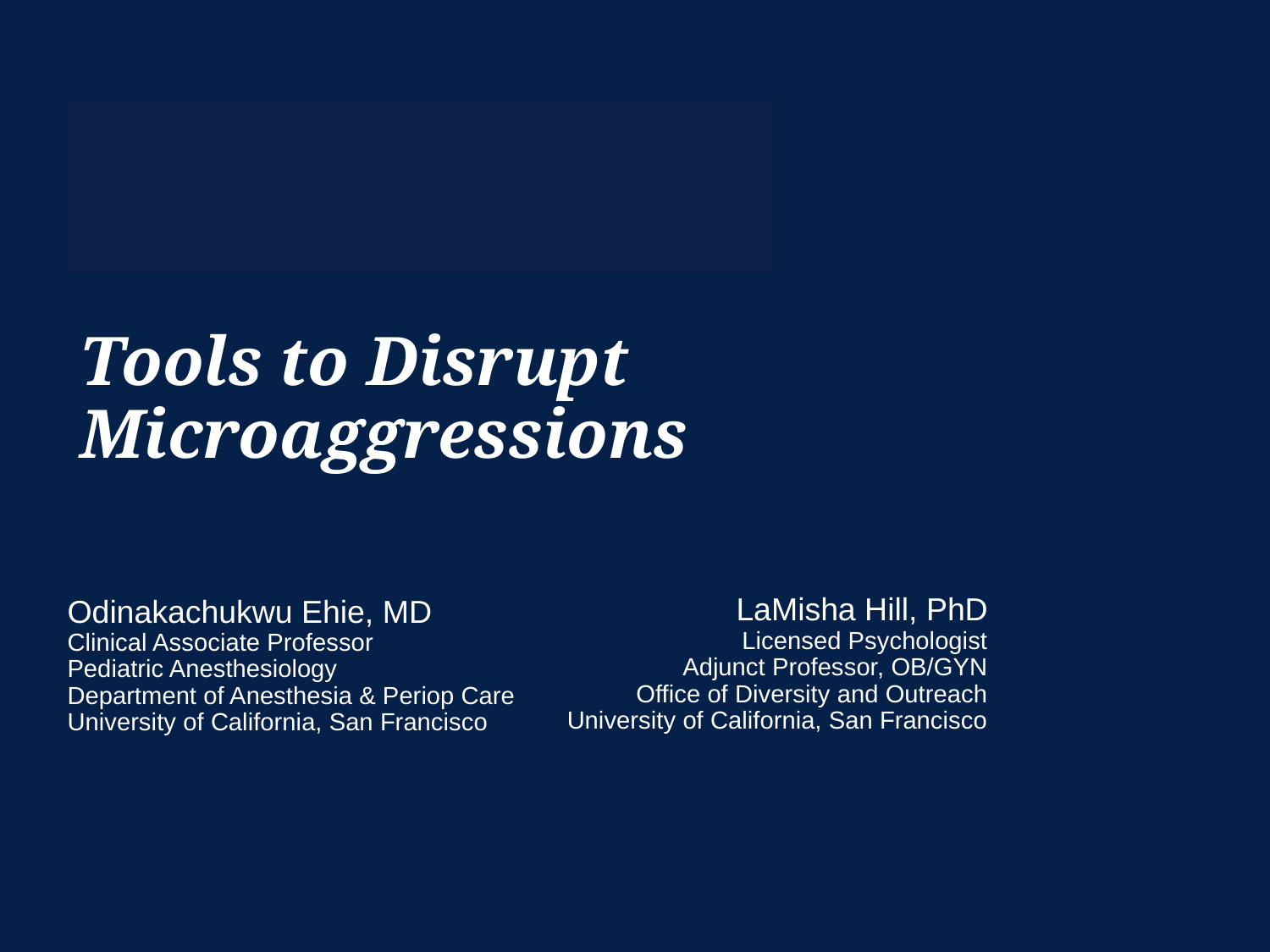

Tools to Disrupt Microaggressions
LaMisha Hill, PhD
Licensed Psychologist
Adjunct Professor, OB/GYN
Office of Diversity and Outreach
University of California, San Francisco
Odinakachukwu Ehie, MD
Clinical Associate Professor
Pediatric Anesthesiology
Department of Anesthesia & Periop Care
University of California, San Francisco

## Slide 2
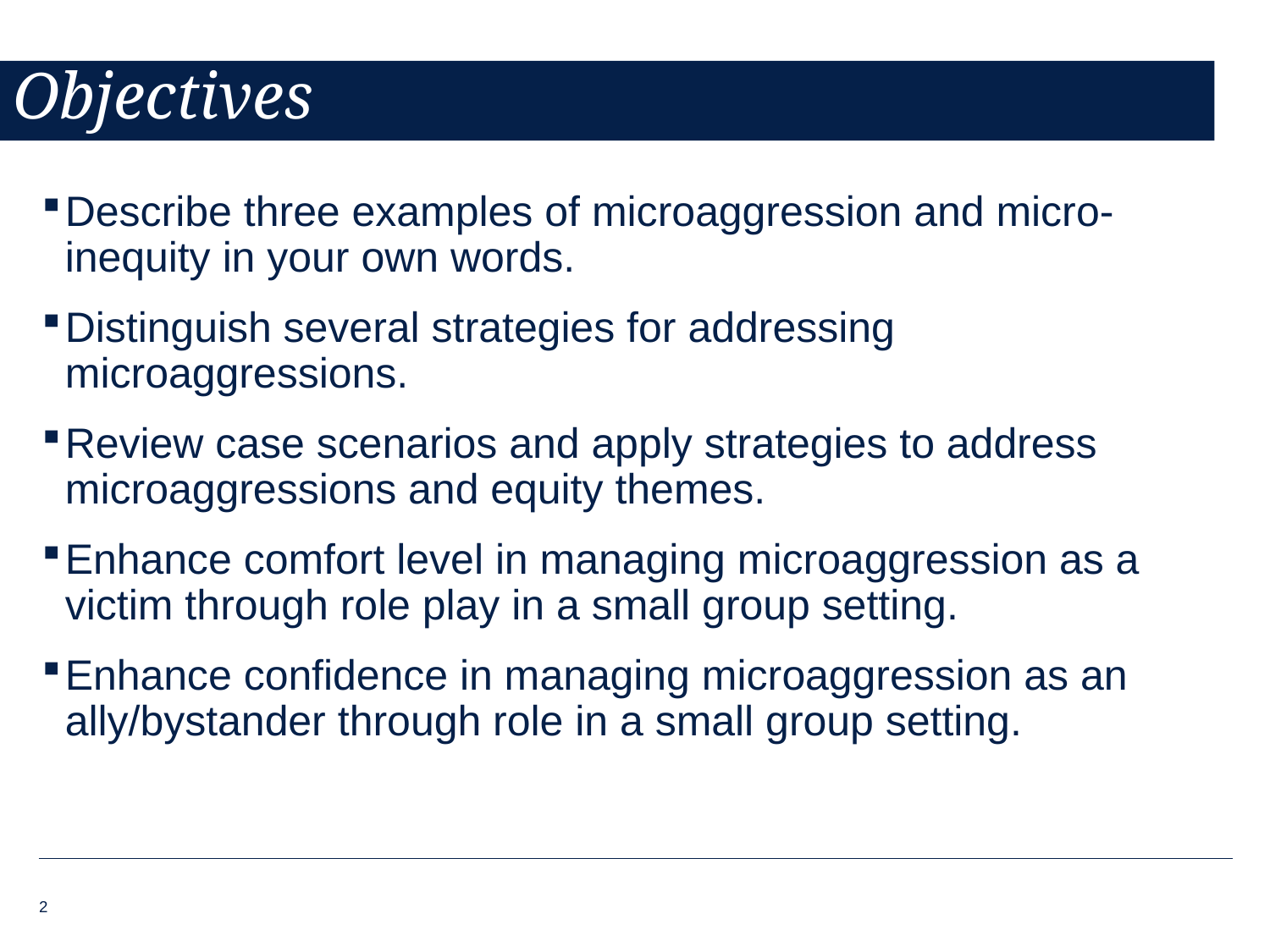

# Objectives
Describe three examples of microaggression and micro-inequity in your own words.
Distinguish several strategies for addressing microaggressions.
Review case scenarios and apply strategies to address microaggressions and equity themes.
Enhance comfort level in managing microaggression as a victim through role play in a small group setting.
Enhance confidence in managing microaggression as an ally/bystander through role in a small group setting.
1

## Slide 3
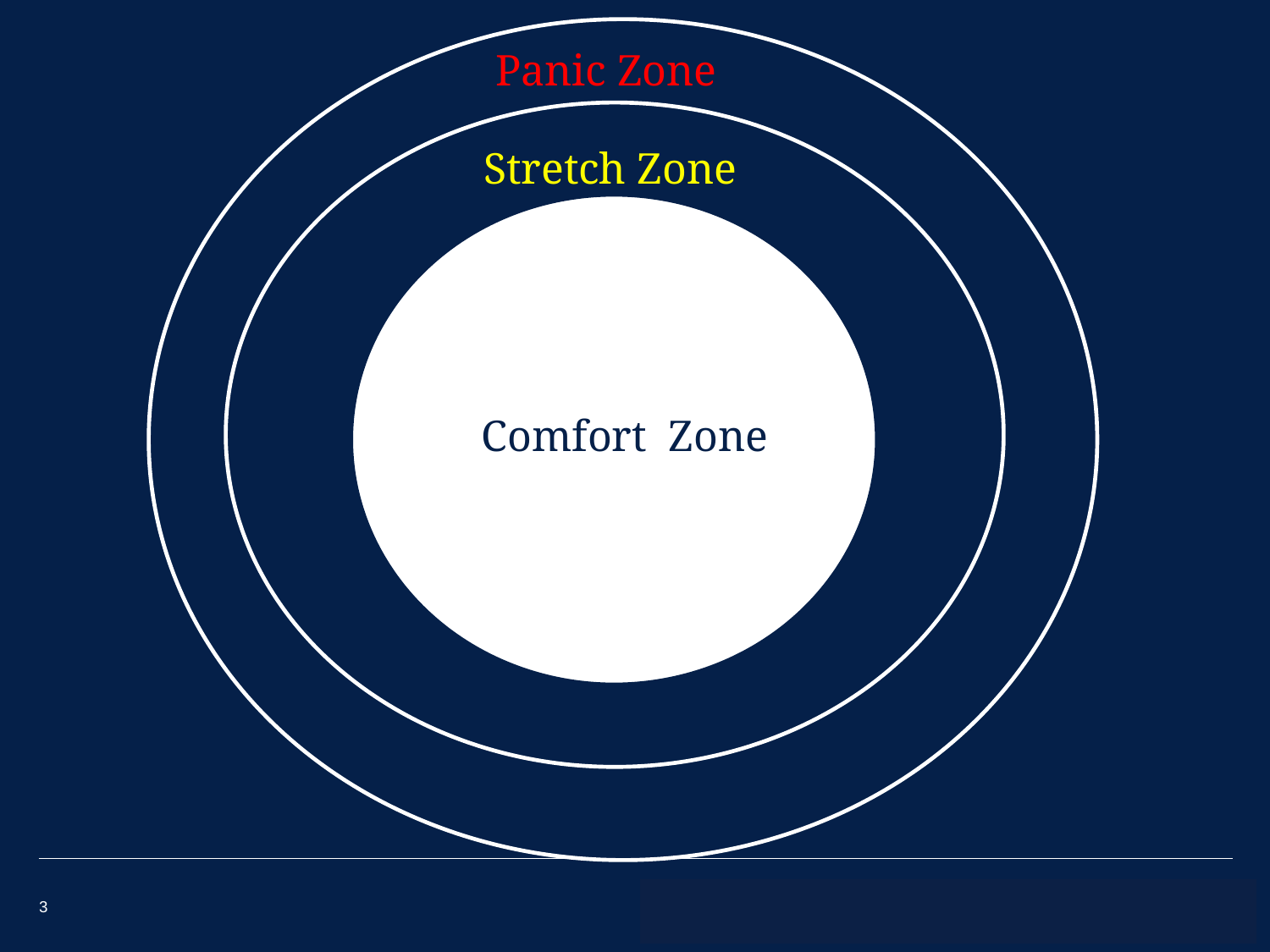

Panic Zone
Stretch Zone
Comfort Zone
2

## Slide 4
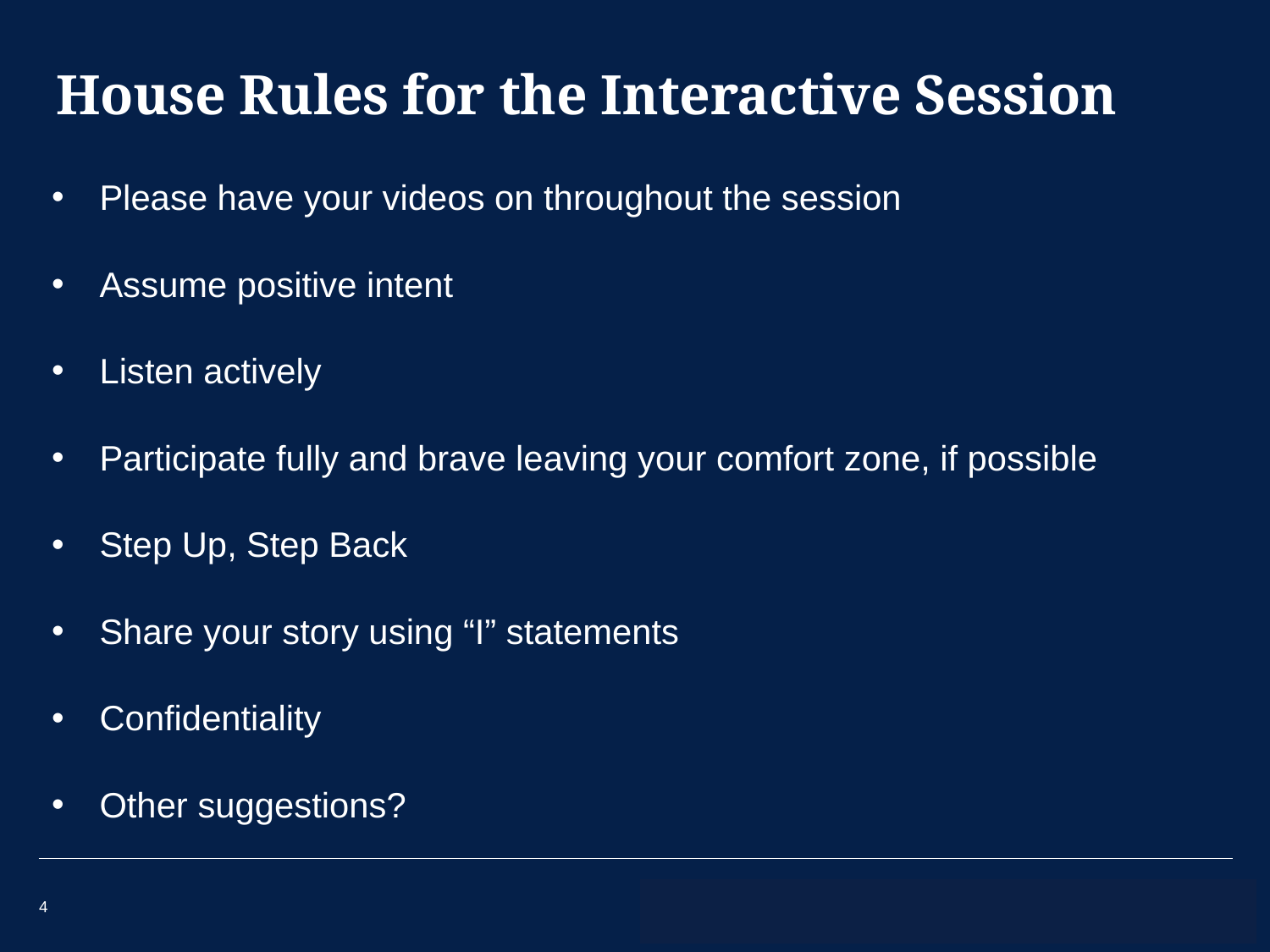

# House Rules for the Interactive Session
Please have your videos on throughout the session
Assume positive intent
Listen actively
Participate fully and brave leaving your comfort zone, if possible
Step Up, Step Back
Share your story using “I” statements
Confidentiality
Other suggestions?
3

## Slide 5
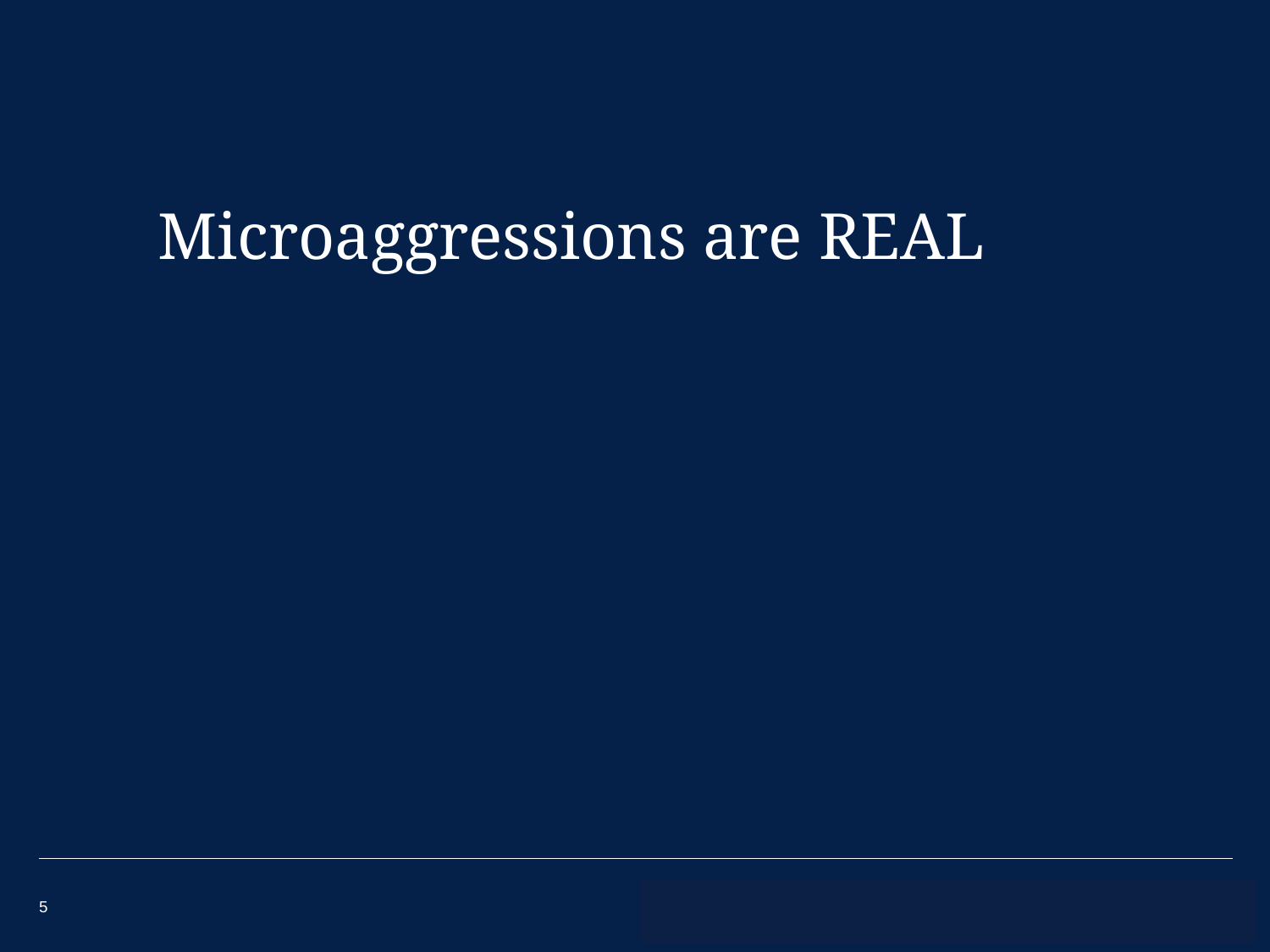

# Microaggressions are REAL
4

## Slide 6
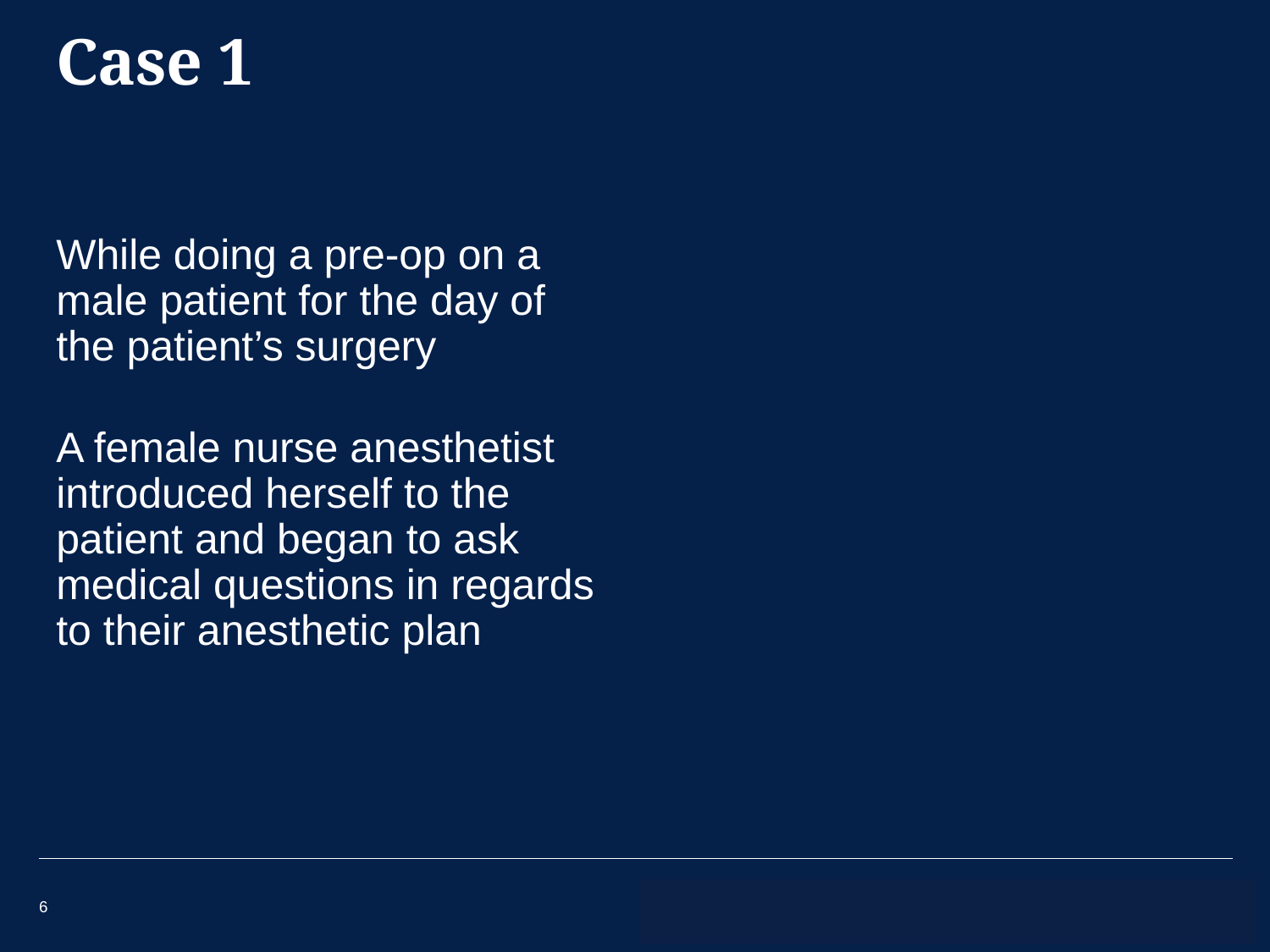

# Case 1
While doing a pre-op on a male patient for the day of the patient’s surgery
A female nurse anesthetist introduced herself to the patient and began to ask medical questions in regards to their anesthetic plan
5

## Slide 7
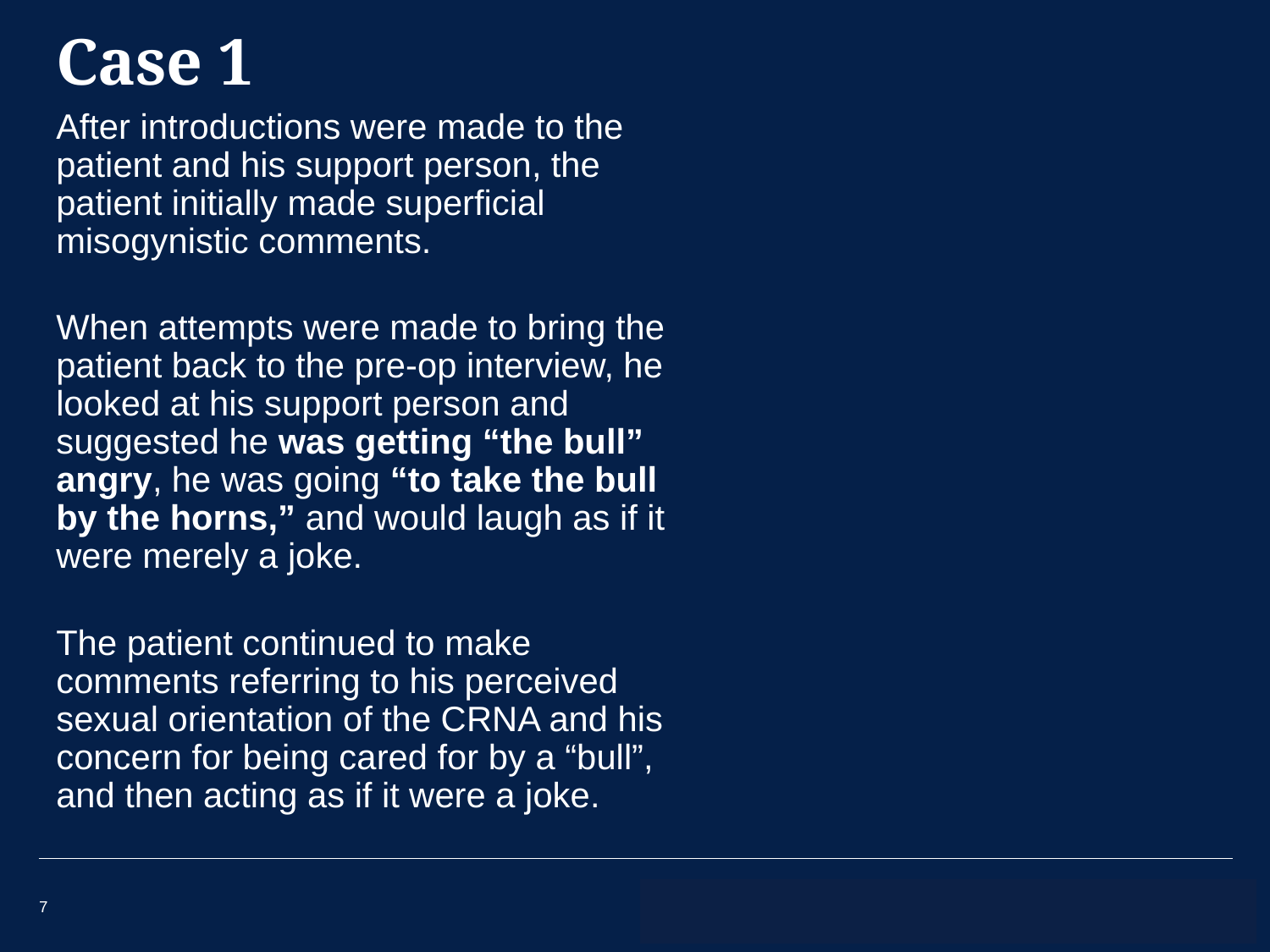

# Case 1
After introductions were made to the patient and his support person, the patient initially made superficial misogynistic comments.
When attempts were made to bring the patient back to the pre-op interview, he looked at his support person and suggested he was getting “the bull” angry, he was going “to take the bull by the horns,” and would laugh as if it were merely a joke.
The patient continued to make comments referring to his perceived sexual orientation of the CRNA and his concern for being cared for by a “bull”, and then acting as if it were a joke.
6

## Slide 8
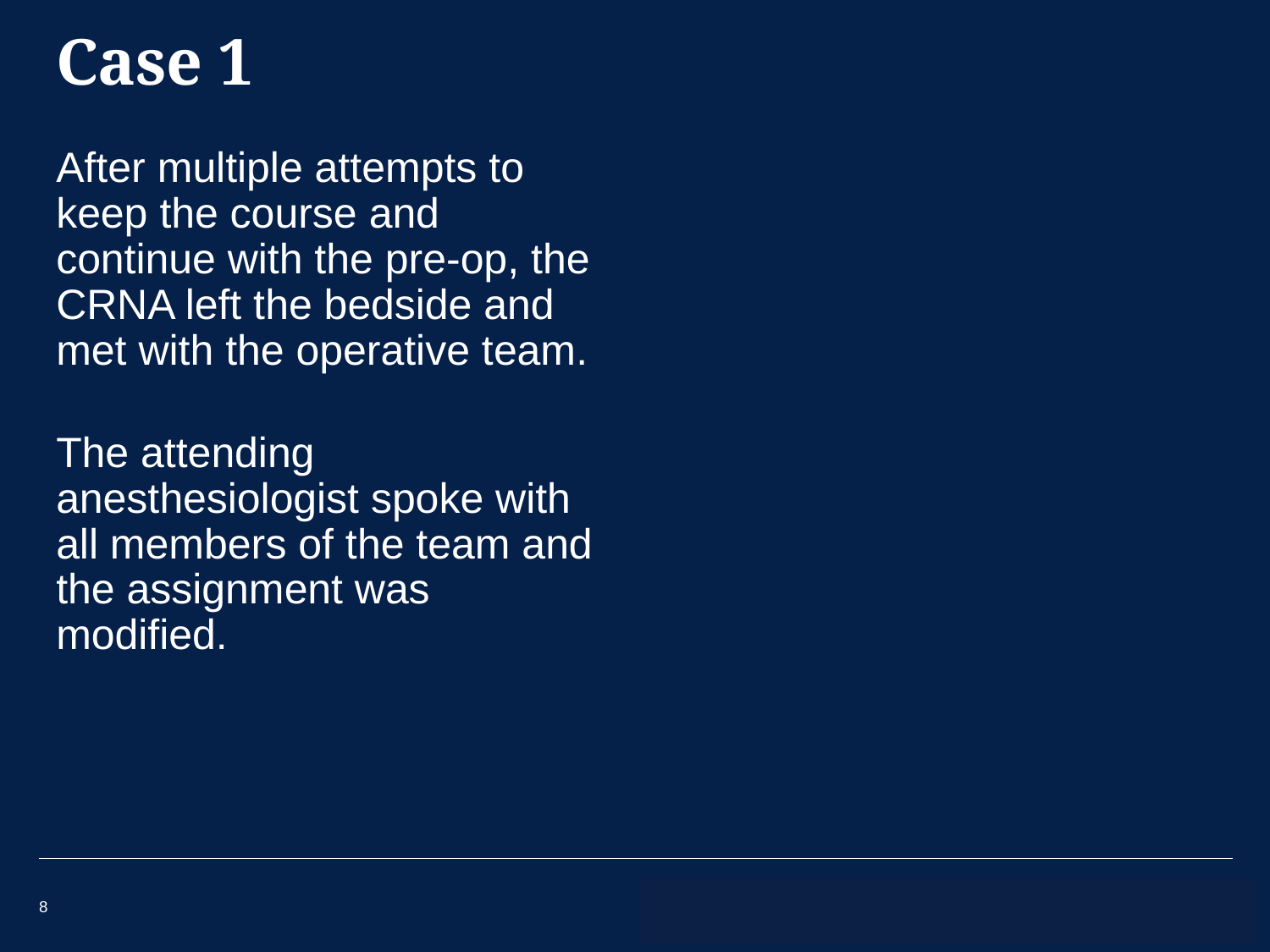

# Case 1
After multiple attempts to keep the course and continue with the pre-op, the CRNA left the bedside and met with the operative team.
The attending anesthesiologist spoke with all members of the team and the assignment was modified.
7

## Slide 9
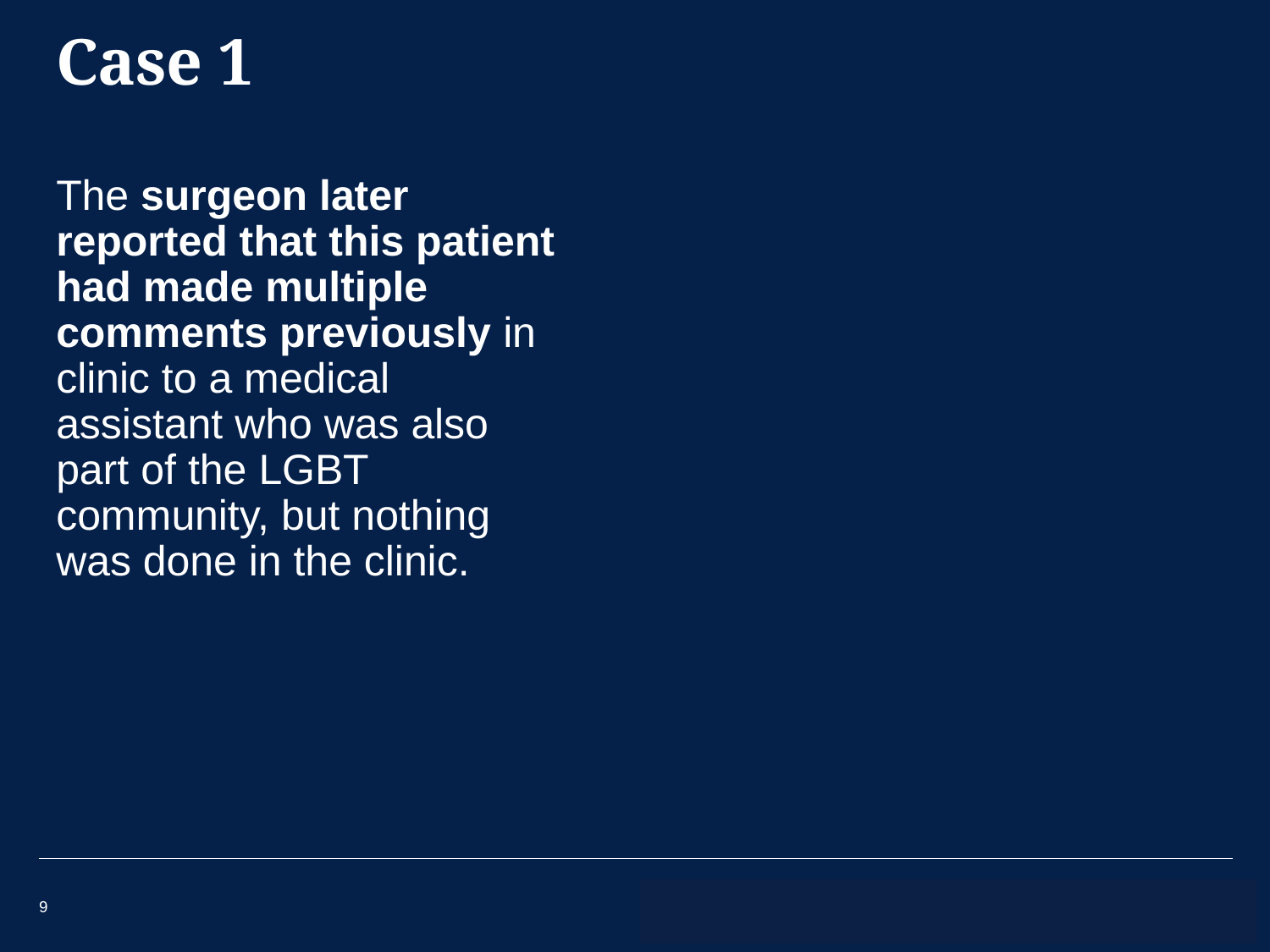

# Case 1
The surgeon later reported that this patient had made multiple comments previously in clinic to a medical assistant who was also part of the LGBT community, but nothing was done in the clinic.
8

## Slide 10
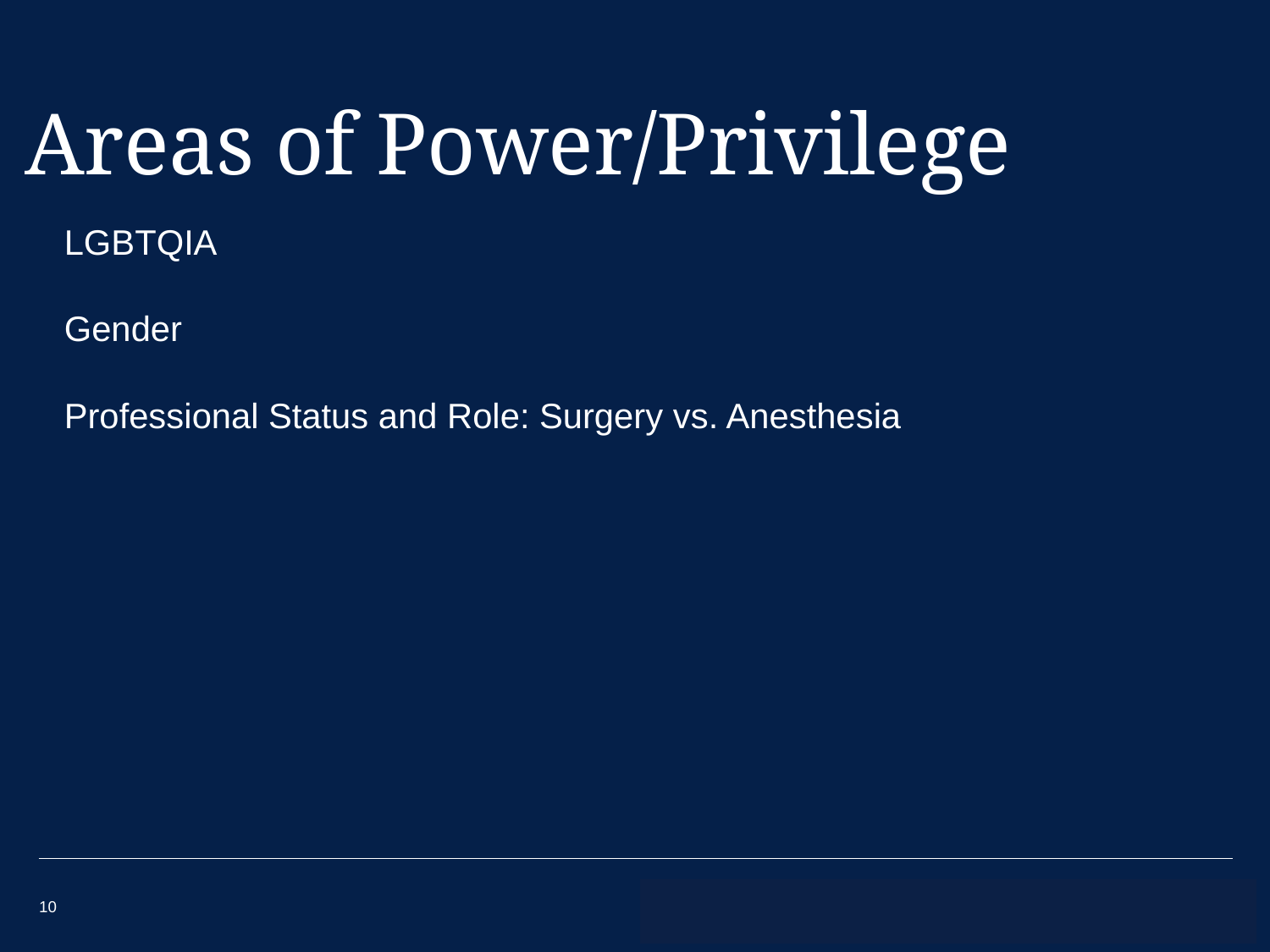

# Areas of Power/Privilege
LGBTQIA
Gender
Professional Status and Role: Surgery vs. Anesthesia
9

## Slide 11
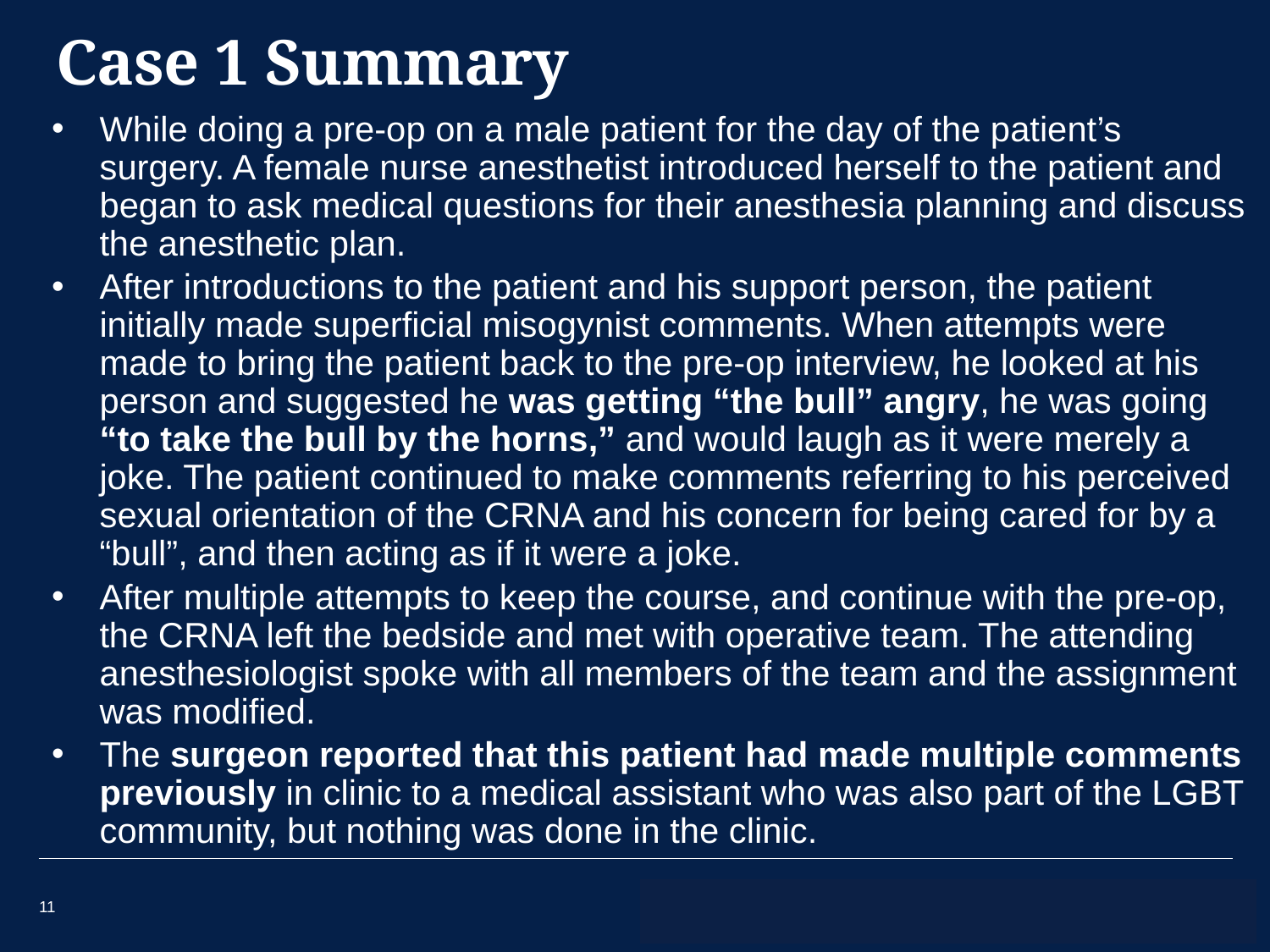

# Case 1 Summary
While doing a pre-op on a male patient for the day of the patient’s surgery. A female nurse anesthetist introduced herself to the patient and began to ask medical questions for their anesthesia planning and discuss the anesthetic plan.
After introductions to the patient and his support person, the patient initially made superficial misogynist comments. When attempts were made to bring the patient back to the pre-op interview, he looked at his person and suggested he was getting “the bull” angry, he was going “to take the bull by the horns,” and would laugh as it were merely a joke. The patient continued to make comments referring to his perceived sexual orientation of the CRNA and his concern for being cared for by a “bull”, and then acting as if it were a joke.
After multiple attempts to keep the course, and continue with the pre-op, the CRNA left the bedside and met with operative team. The attending anesthesiologist spoke with all members of the team and the assignment was modified.
The surgeon reported that this patient had made multiple comments previously in clinic to a medical assistant who was also part of the LGBT community, but nothing was done in the clinic.
10

## Slide 12
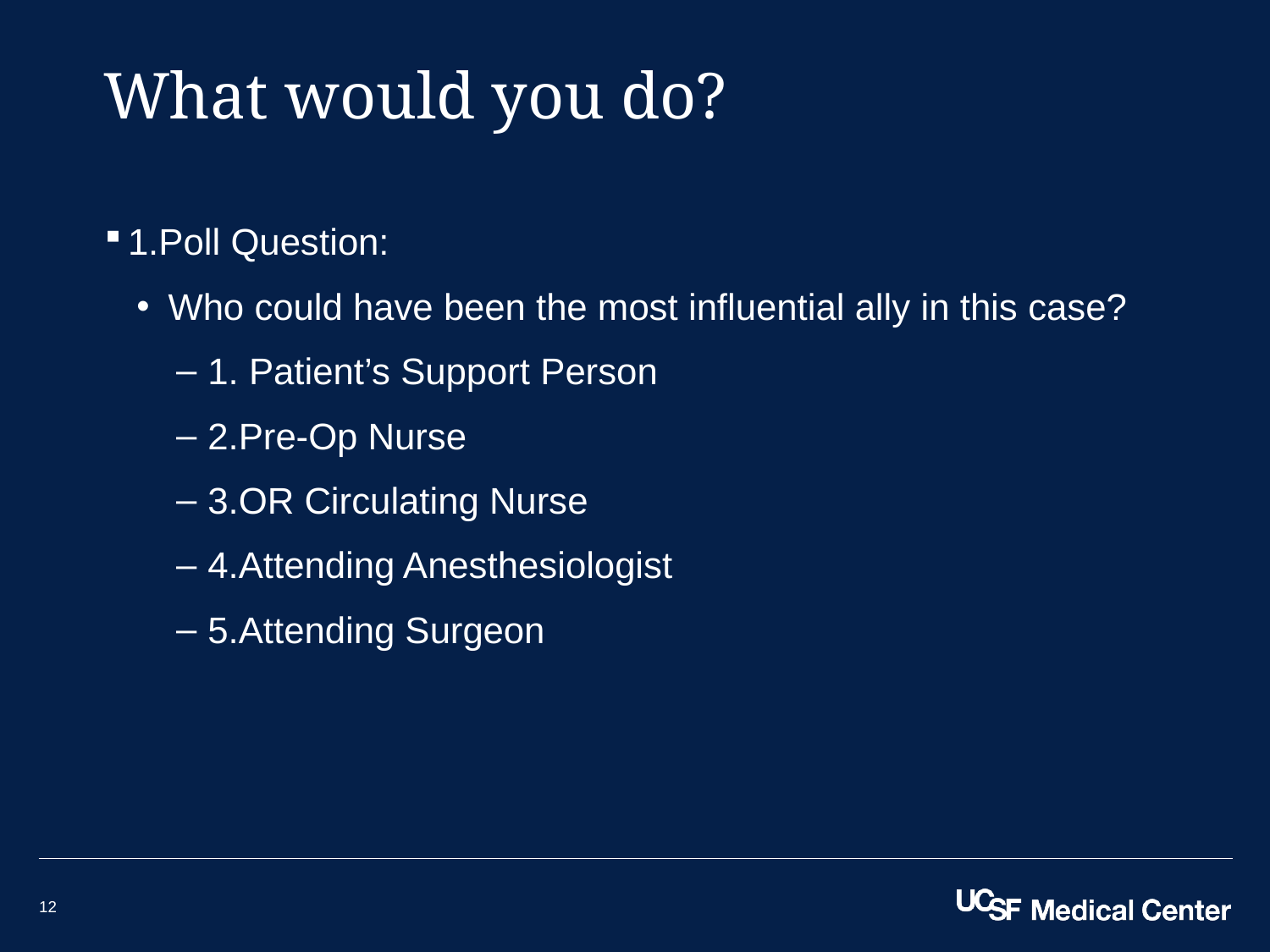

# What would you do?
1.Poll Question:
Who could have been the most influential ally in this case?
1. Patient’s Support Person
2.Pre-Op Nurse
3.OR Circulating Nurse
4.Attending Anesthesiologist
5.Attending Surgeon
11

## Slide 13
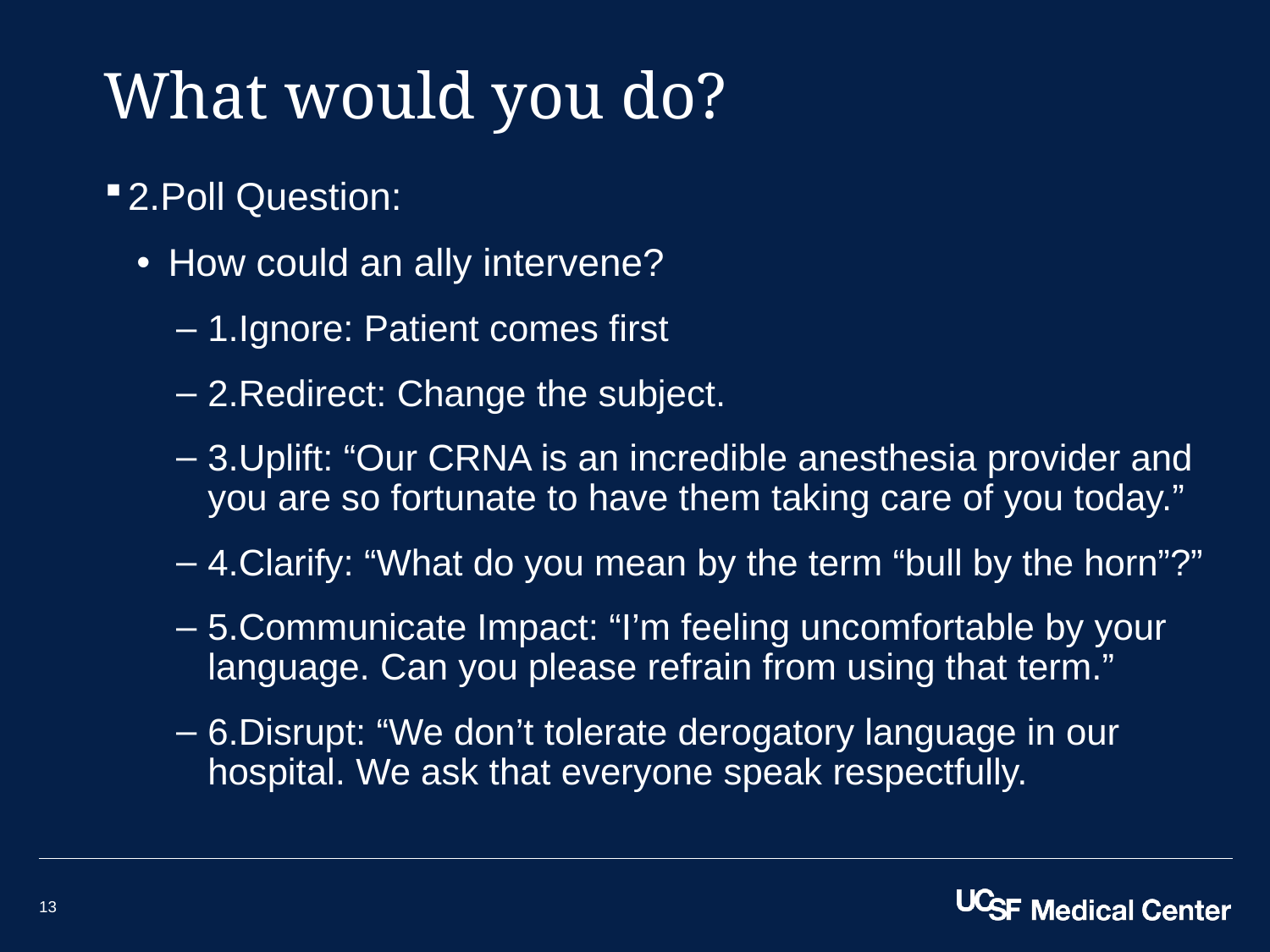

# What would you do?
2.Poll Question:
How could an ally intervene?
1.Ignore: Patient comes first
2.Redirect: Change the subject.
3.Uplift: “Our CRNA is an incredible anesthesia provider and you are so fortunate to have them taking care of you today.”
4.Clarify: “What do you mean by the term “bull by the horn”?”
5.Communicate Impact: “I’m feeling uncomfortable by your language. Can you please refrain from using that term.”
6.Disrupt: “We don’t tolerate derogatory language in our hospital. We ask that everyone speak respectfully.
12

## Slide 14
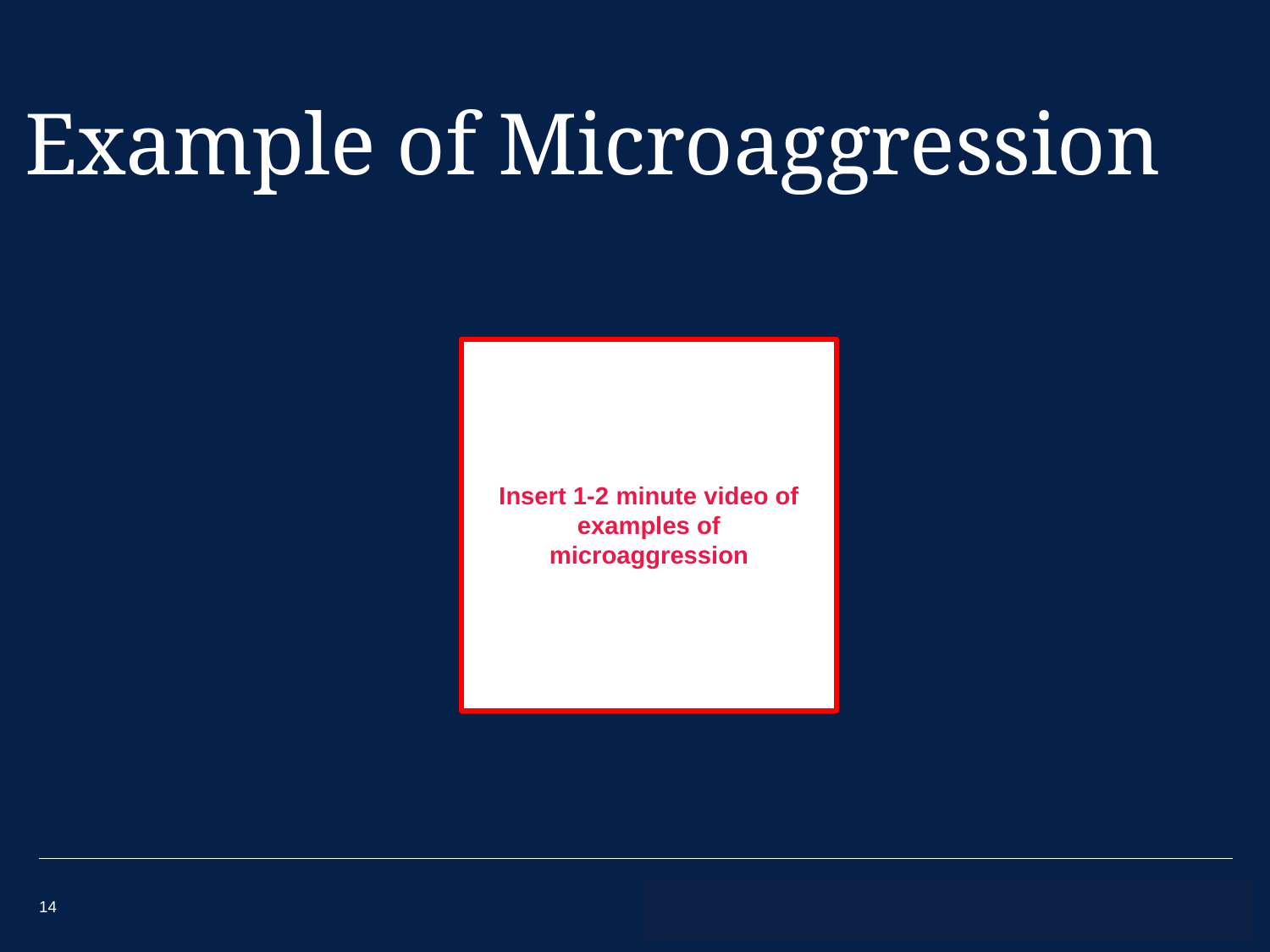

# Example of Microaggression
Insert 1-2 minute video of examples of microaggression
13

## Slide 15
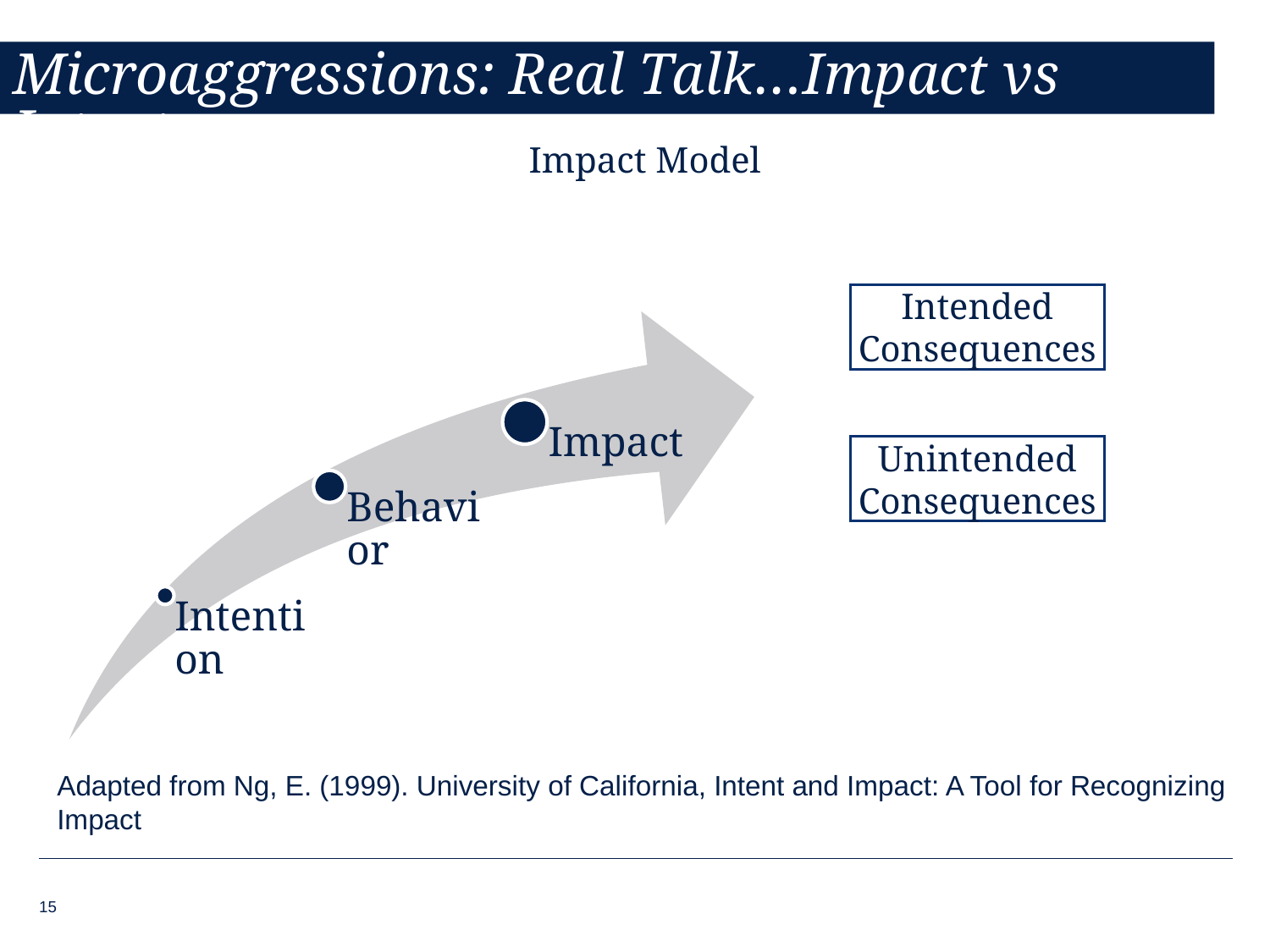

# Microaggressions: Real Talk…Impact vs Intent
Impact Model
Intended Consequences
Unintended Consequences
Adapted from Ng, E. (1999). University of California, Intent and Impact: A Tool for Recognizing Impact
14

## Slide 16
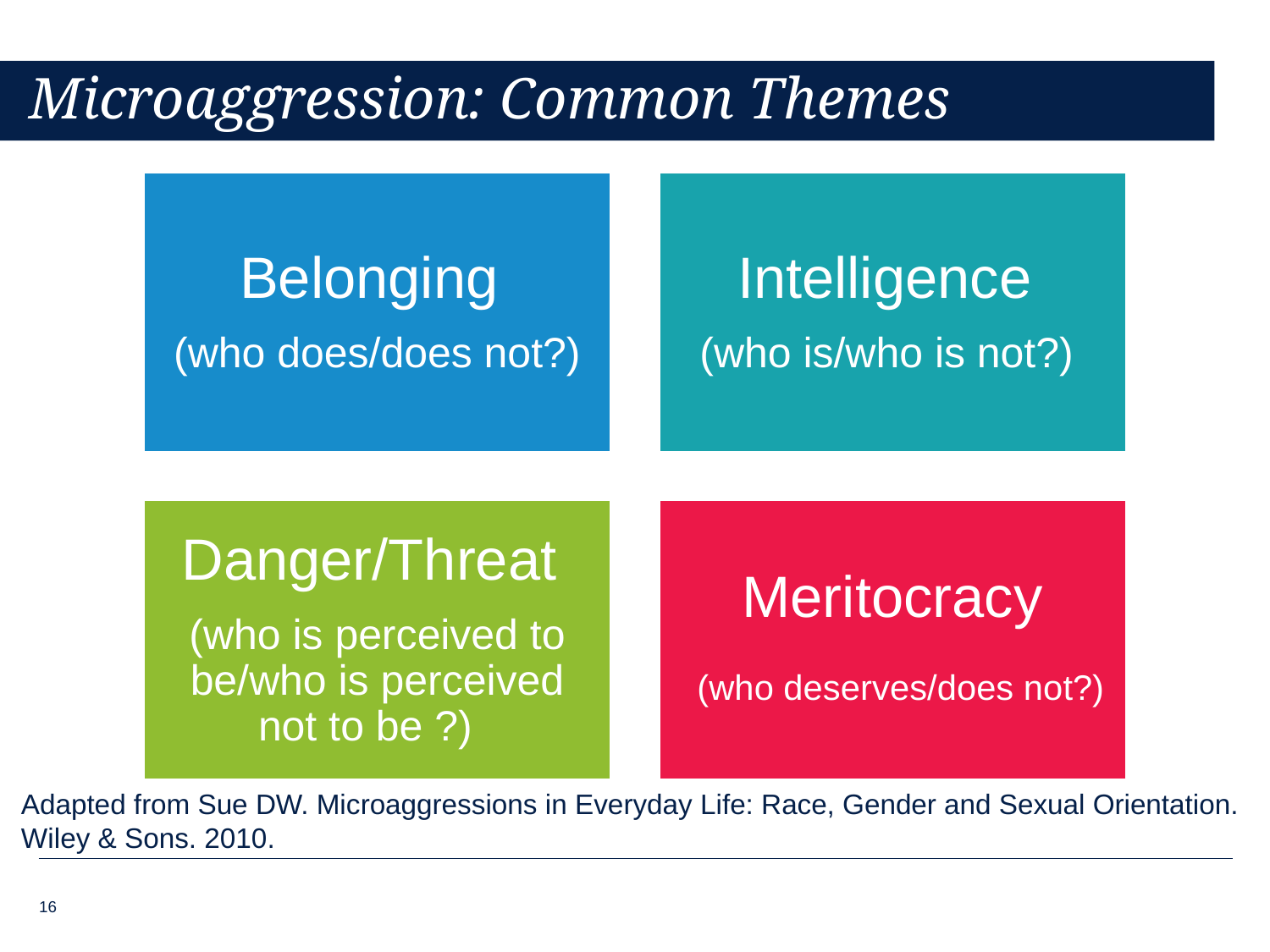

# Microaggression: Common Themes
Adapted from Sue DW. Microaggressions in Everyday Life: Race, Gender and Sexual Orientation. Wiley & Sons. 2010.
15

## Slide 17
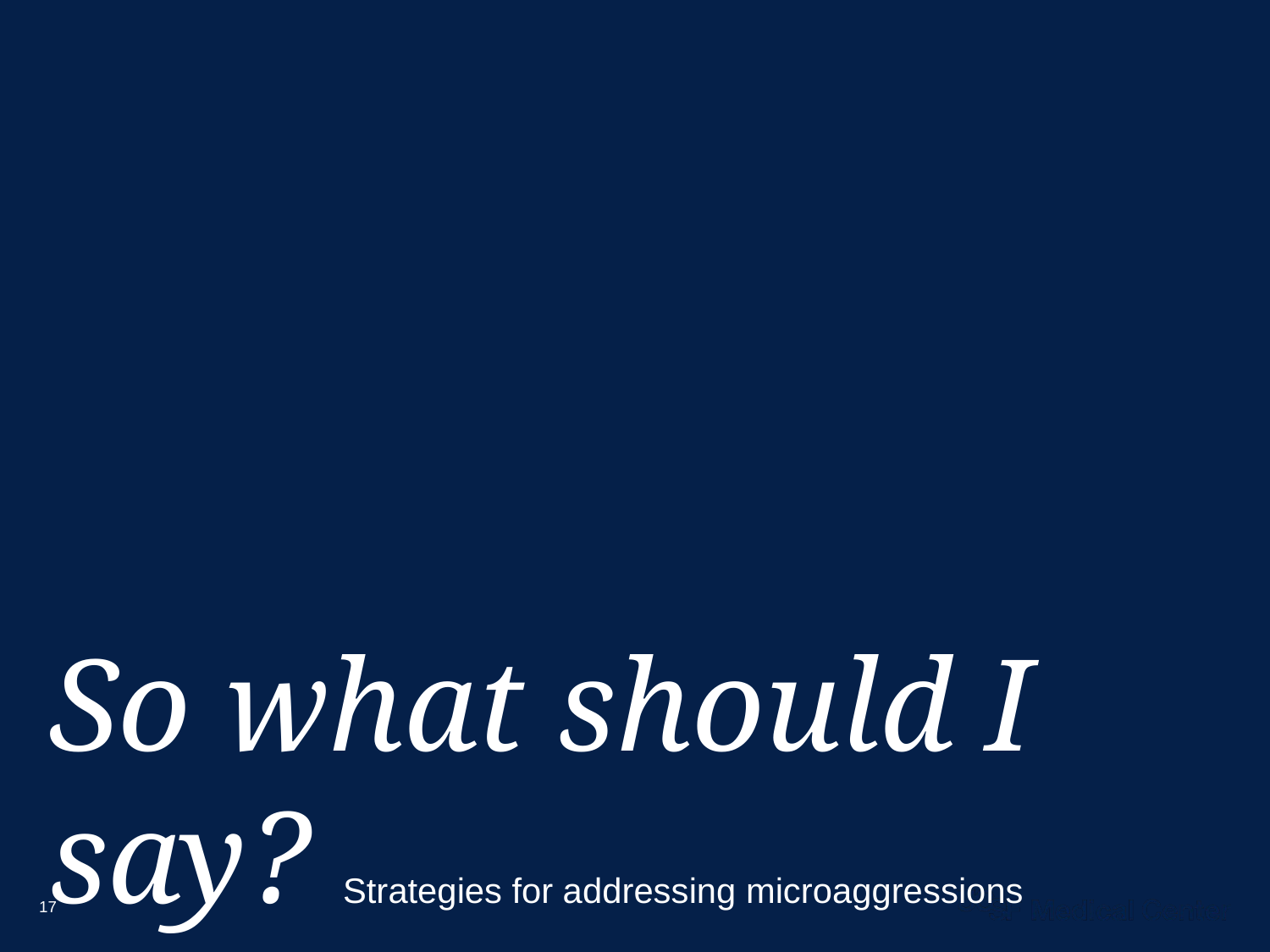

So what should I say? Strategies for addressing microaggressions
16

## Slide 18
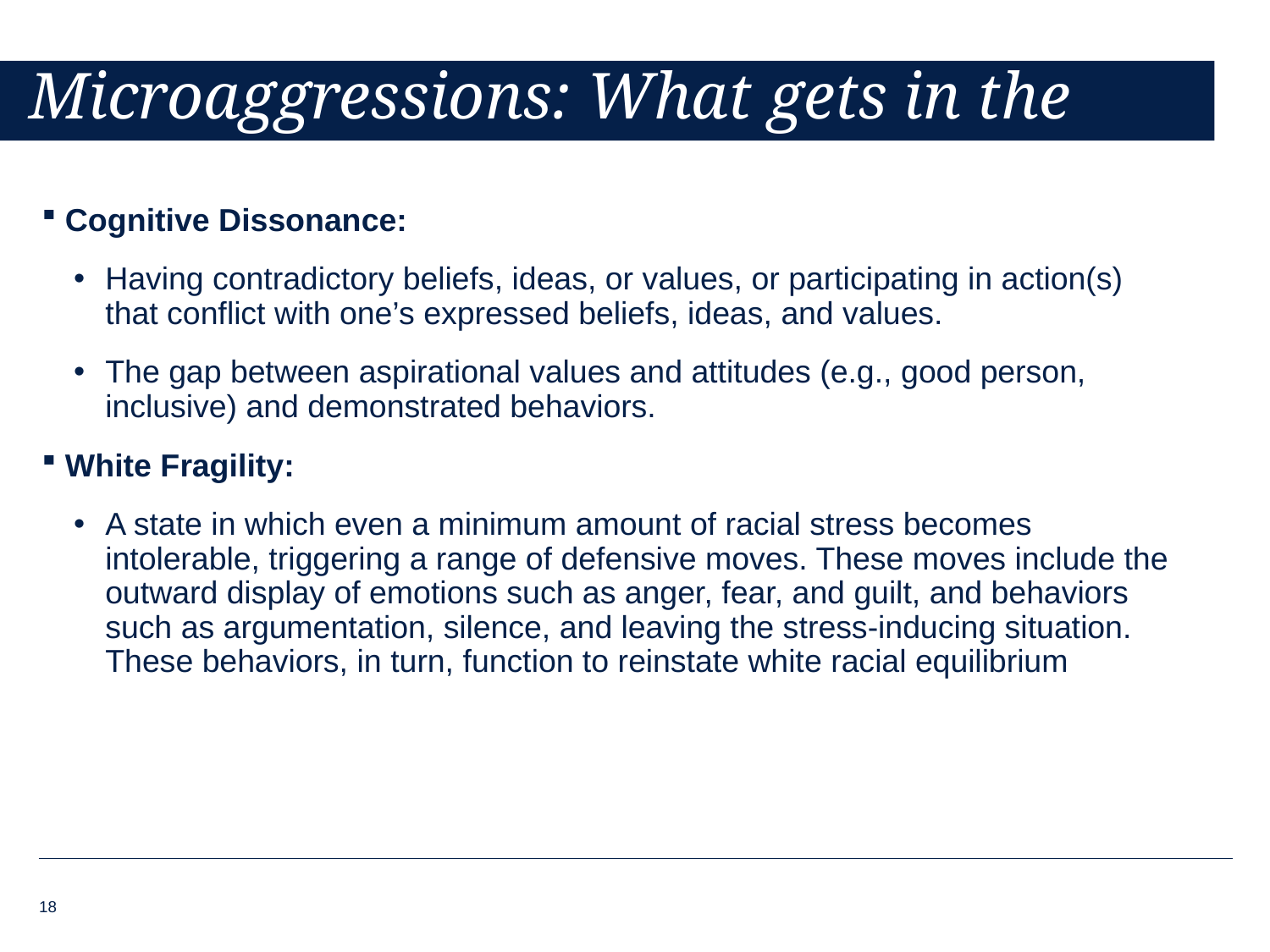

# Microaggressions: What gets in the way…
Cognitive Dissonance:
Having contradictory beliefs, ideas, or values, or participating in action(s) that conflict with one’s expressed beliefs, ideas, and values.
The gap between aspirational values and attitudes (e.g., good person, inclusive) and demonstrated behaviors.
White Fragility:
A state in which even a minimum amount of racial stress becomes intolerable, triggering a range of defensive moves. These moves include the outward display of emotions such as anger, fear, and guilt, and behaviors such as argumentation, silence, and leaving the stress-inducing situation. These behaviors, in turn, function to reinstate white racial equilibrium
17

## Slide 19
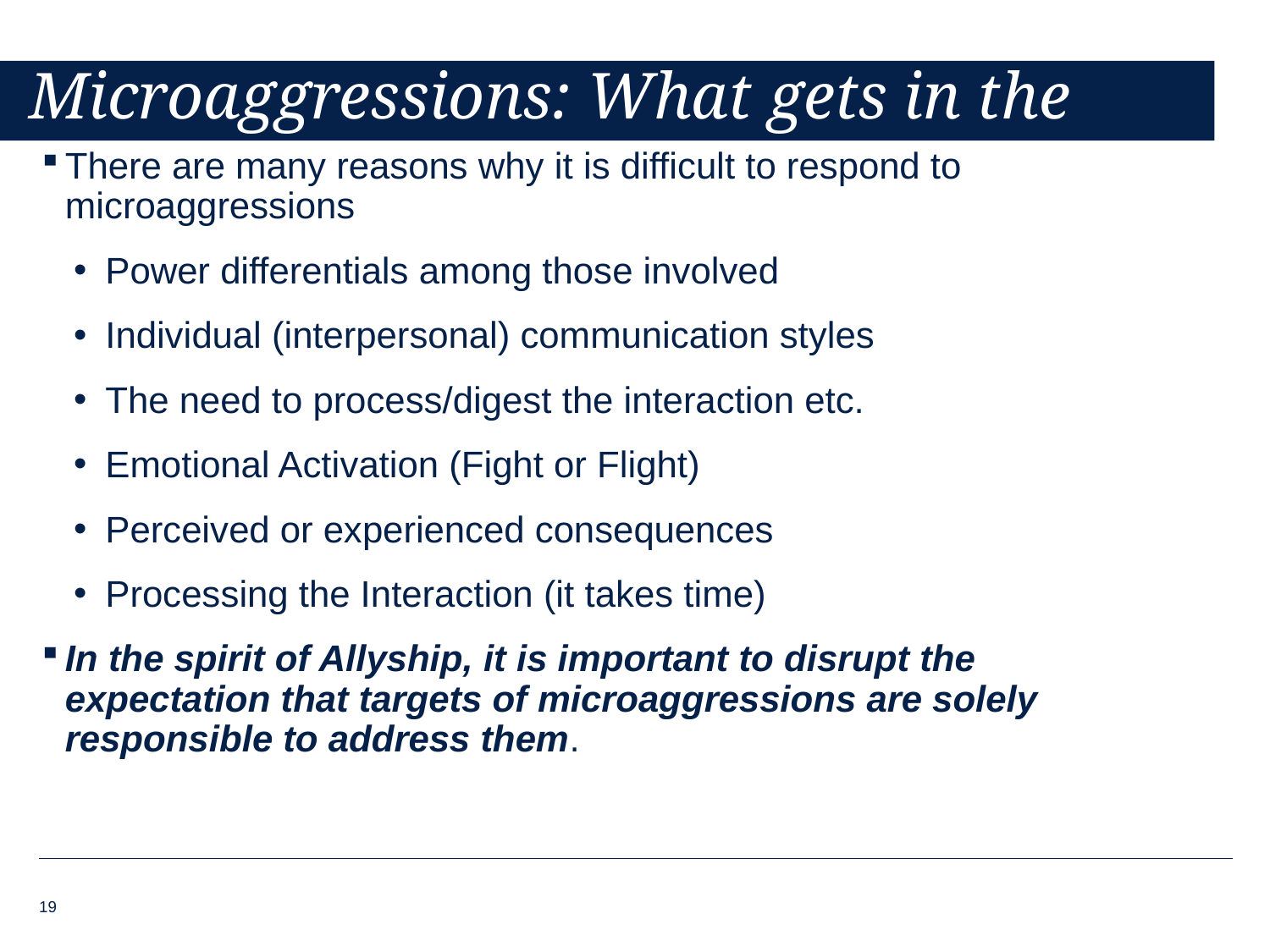

# Microaggressions: What gets in the way…
There are many reasons why it is difficult to respond to microaggressions
Power differentials among those involved
Individual (interpersonal) communication styles
The need to process/digest the interaction etc.
Emotional Activation (Fight or Flight)
Perceived or experienced consequences
Processing the Interaction (it takes time)
In the spirit of Allyship, it is important to disrupt the expectation that targets of microaggressions are solely responsible to address them.
18

## Slide 20
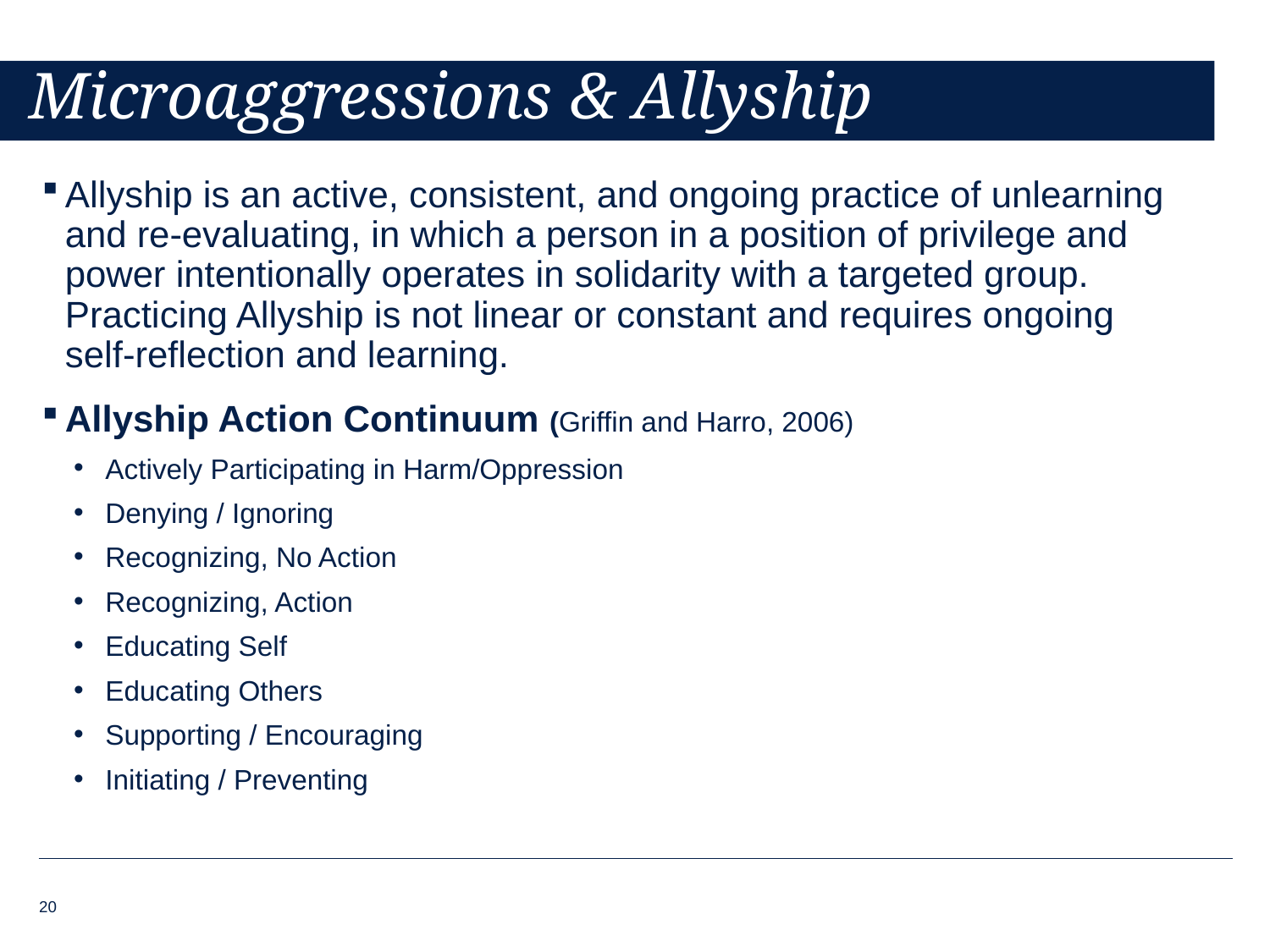

# Microaggressions & Allyship
Allyship is an active, consistent, and ongoing practice of unlearning and re-evaluating, in which a person in a position of privilege and power intentionally operates in solidarity with a targeted group. Practicing Allyship is not linear or constant and requires ongoing self-reflection and learning.
Allyship Action Continuum (Griffin and Harro, 2006)
Actively Participating in Harm/Oppression
Denying / Ignoring
Recognizing, No Action
Recognizing, Action
Educating Self
Educating Others
Supporting / Encouraging
Initiating / Preventing
19

## Slide 21
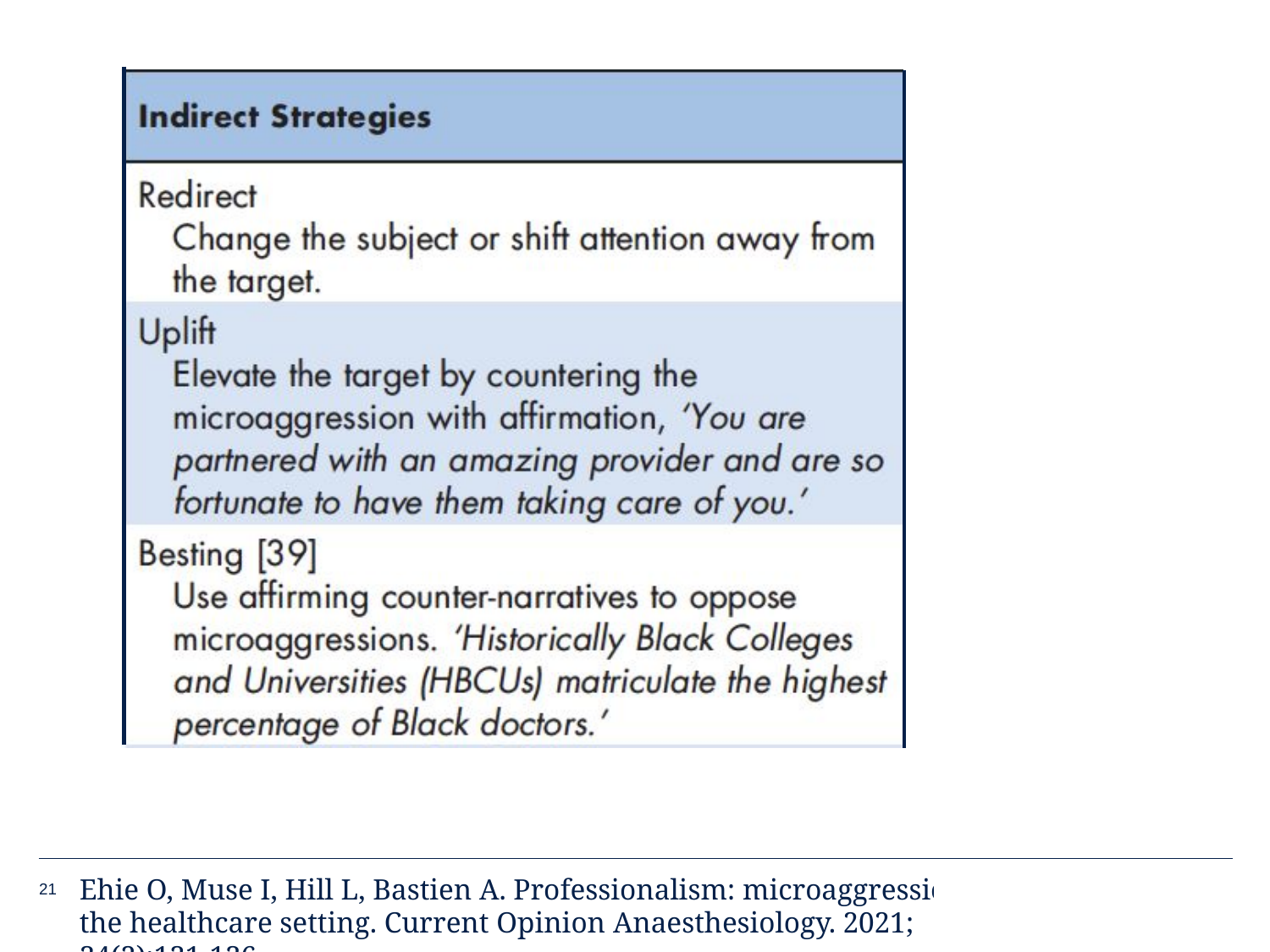

Ehie O, Muse I, Hill L, Bastien A. Professionalism: microaggression in the healthcare setting. Current Opinion Anaesthesiology. 2021; 34(2):131-136.
20

## Slide 22
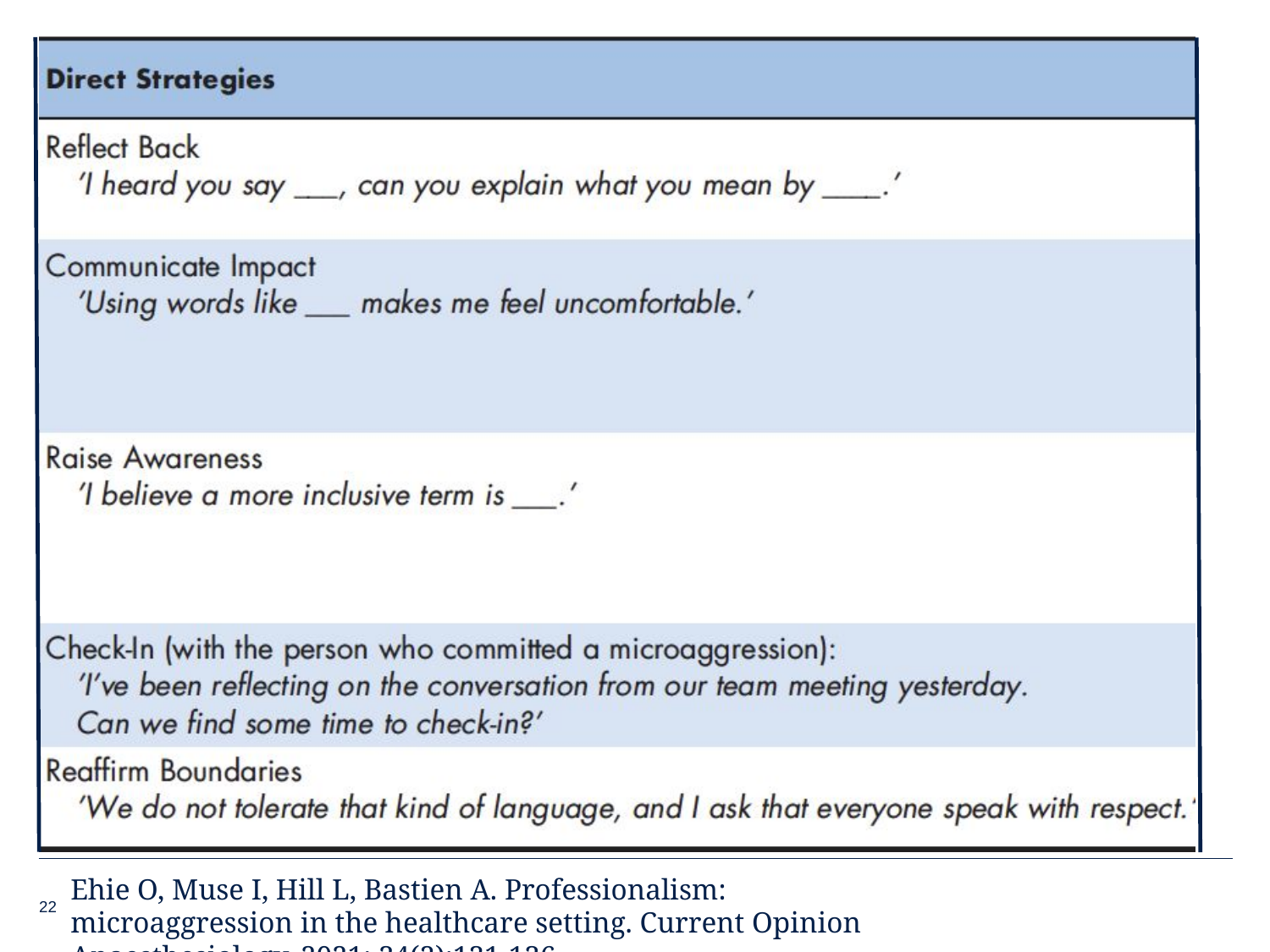

Ehie O, Muse I, Hill L, Bastien A. Professionalism: microaggression in the healthcare setting. Current Opinion Anaesthesiology. 2021; 34(2):131-136.
21

## Slide 23
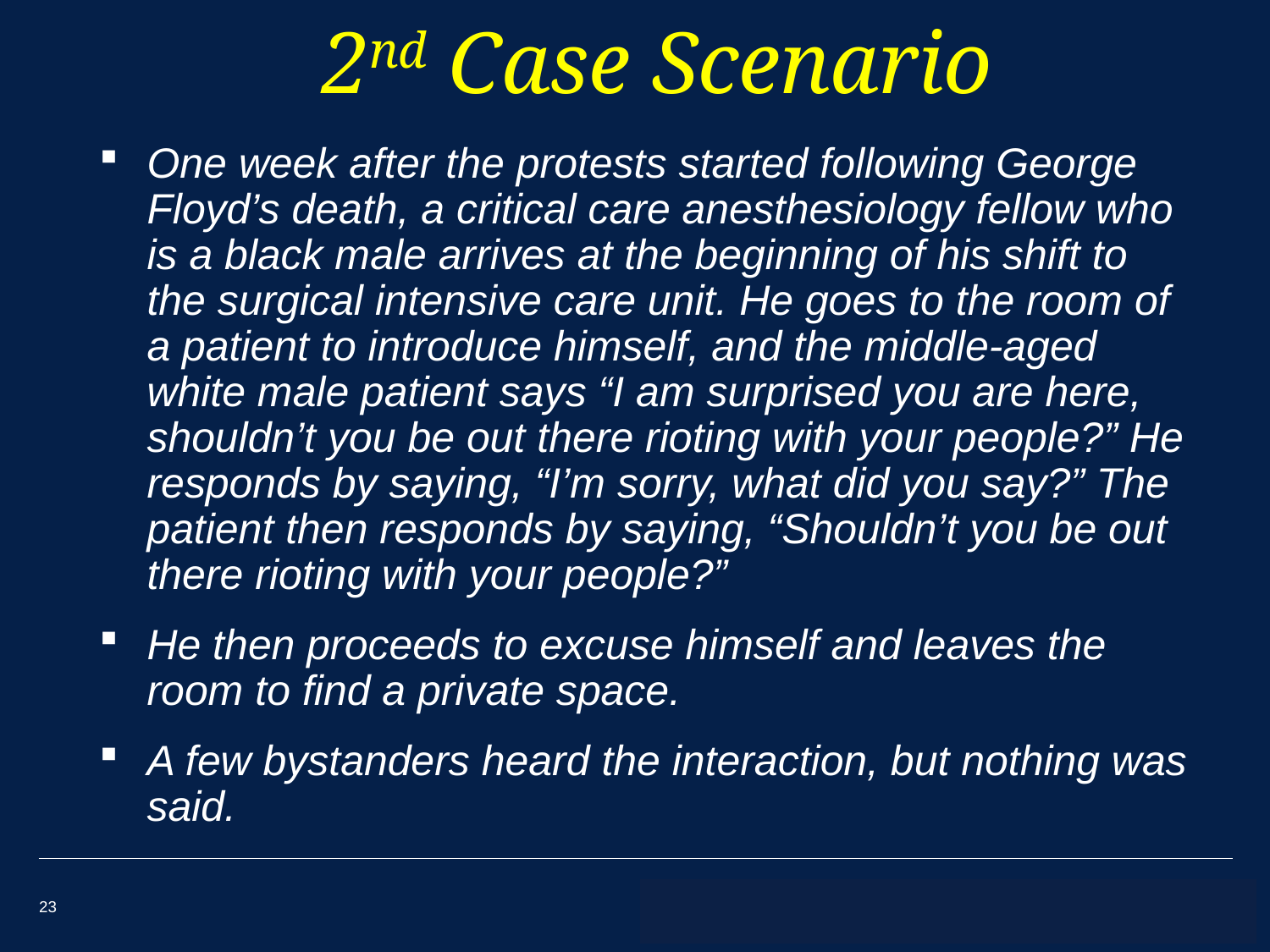

# 2nd Case Scenario
One week after the protests started following George Floyd’s death, a critical care anesthesiology fellow who is a black male arrives at the beginning of his shift to the surgical intensive care unit. He goes to the room of a patient to introduce himself, and the middle-aged white male patient says “I am surprised you are here, shouldn’t you be out there rioting with your people?” He responds by saying, “I’m sorry, what did you say?” The patient then responds by saying, “Shouldn’t you be out there rioting with your people?”
He then proceeds to excuse himself and leaves the room to find a private space.
A few bystanders heard the interaction, but nothing was said.
22

## Slide 24
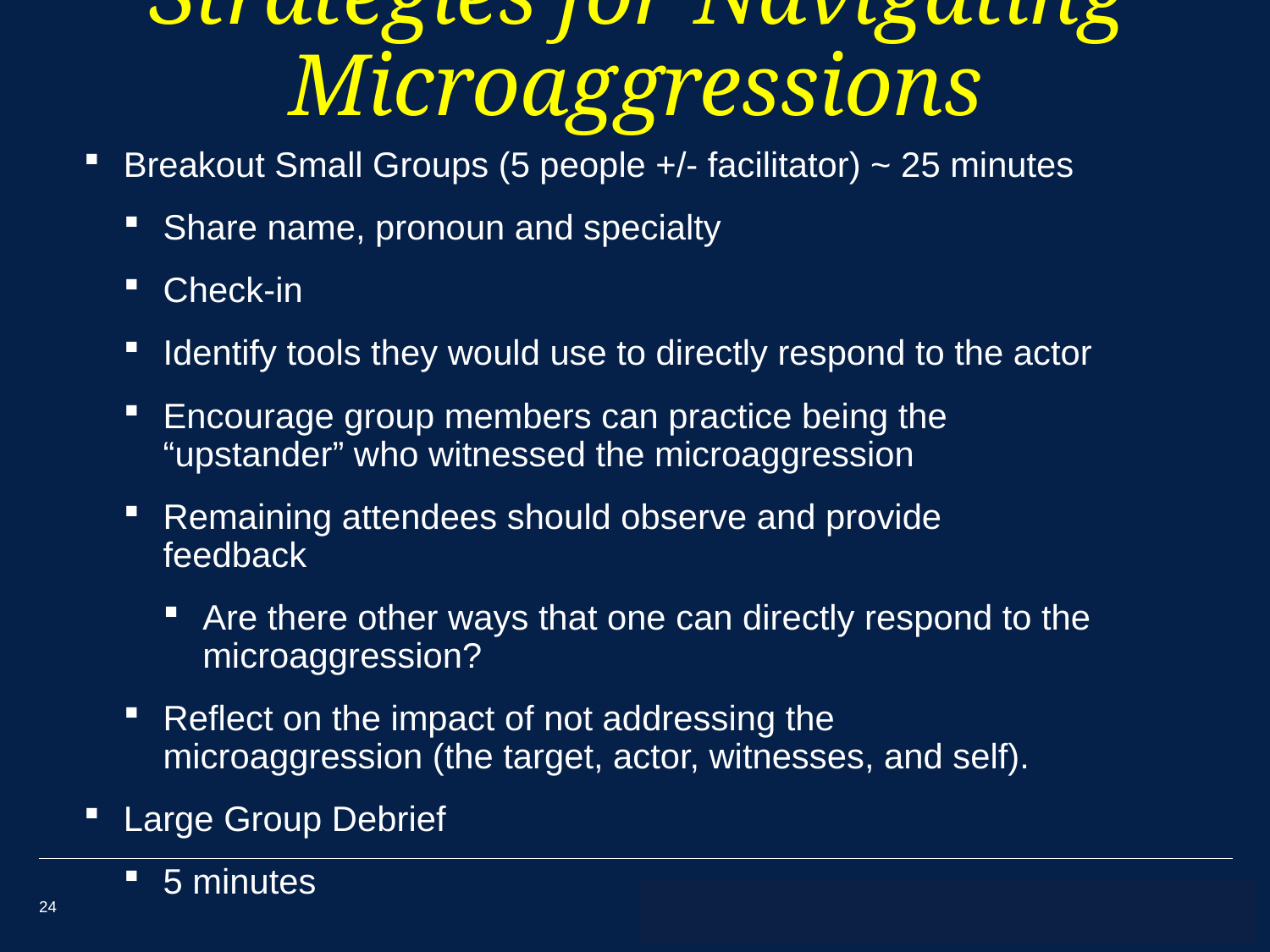

# Strategies for Navigating Microaggressions
Breakout Small Groups (5 people +/- facilitator) ~ 25 minutes
Share name, pronoun and specialty
Check-in
Identify tools they would use to directly respond to the actor
Encourage group members can practice being the “upstander” who witnessed the microaggression
Remaining attendees should observe and provide feedback
Are there other ways that one can directly respond to the microaggression?
Reflect on the impact of not addressing the microaggression (the target, actor, witnesses, and self).
Large Group Debrief
5 minutes
23

## Slide 25
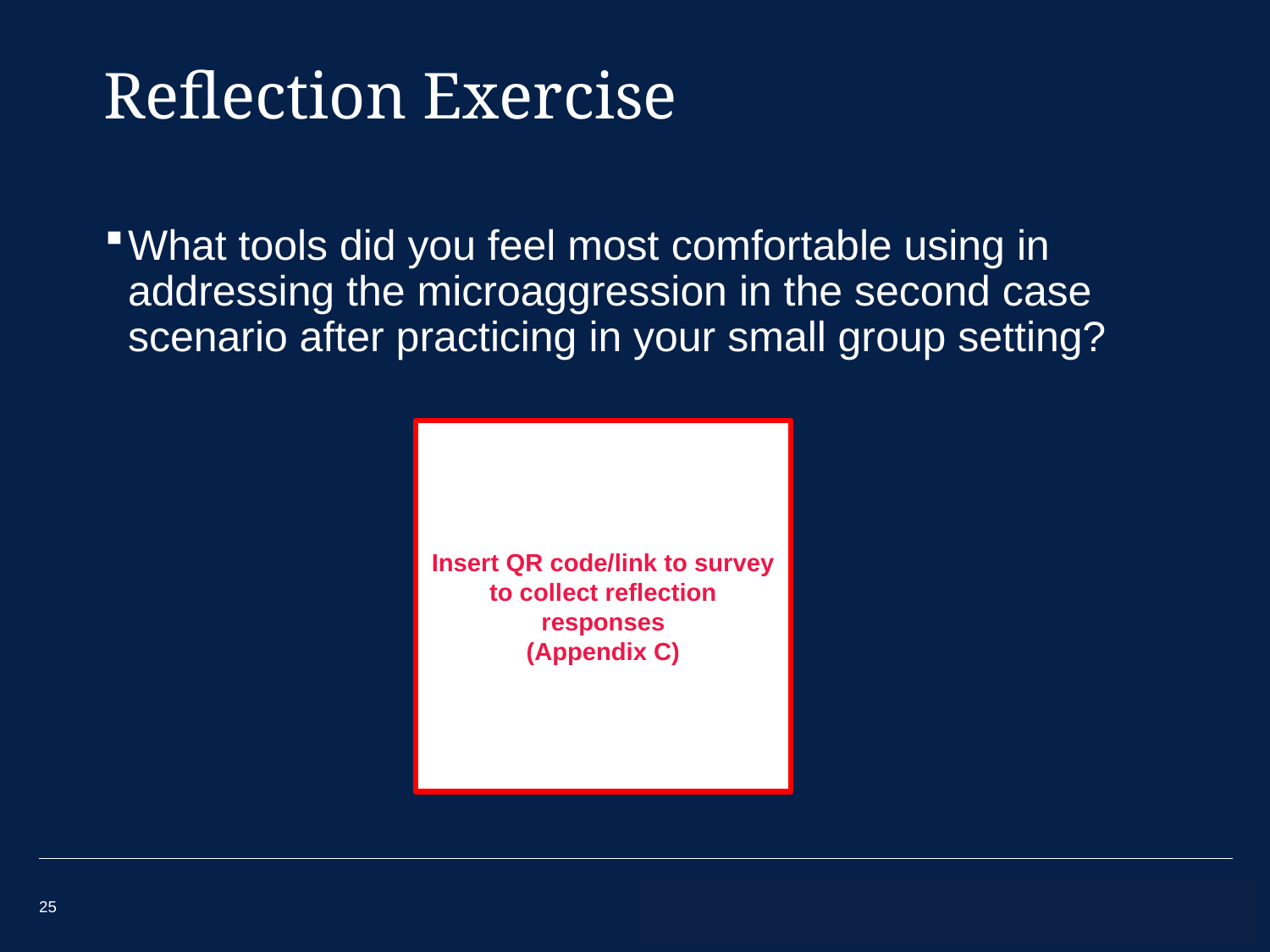

# Reflection Exercise
What tools did you feel most comfortable using in addressing the microaggression in the second case scenario after practicing in your small group setting?
Insert QR code/link to survey to collect reflection responses
(Appendix C)
24

## Slide 26
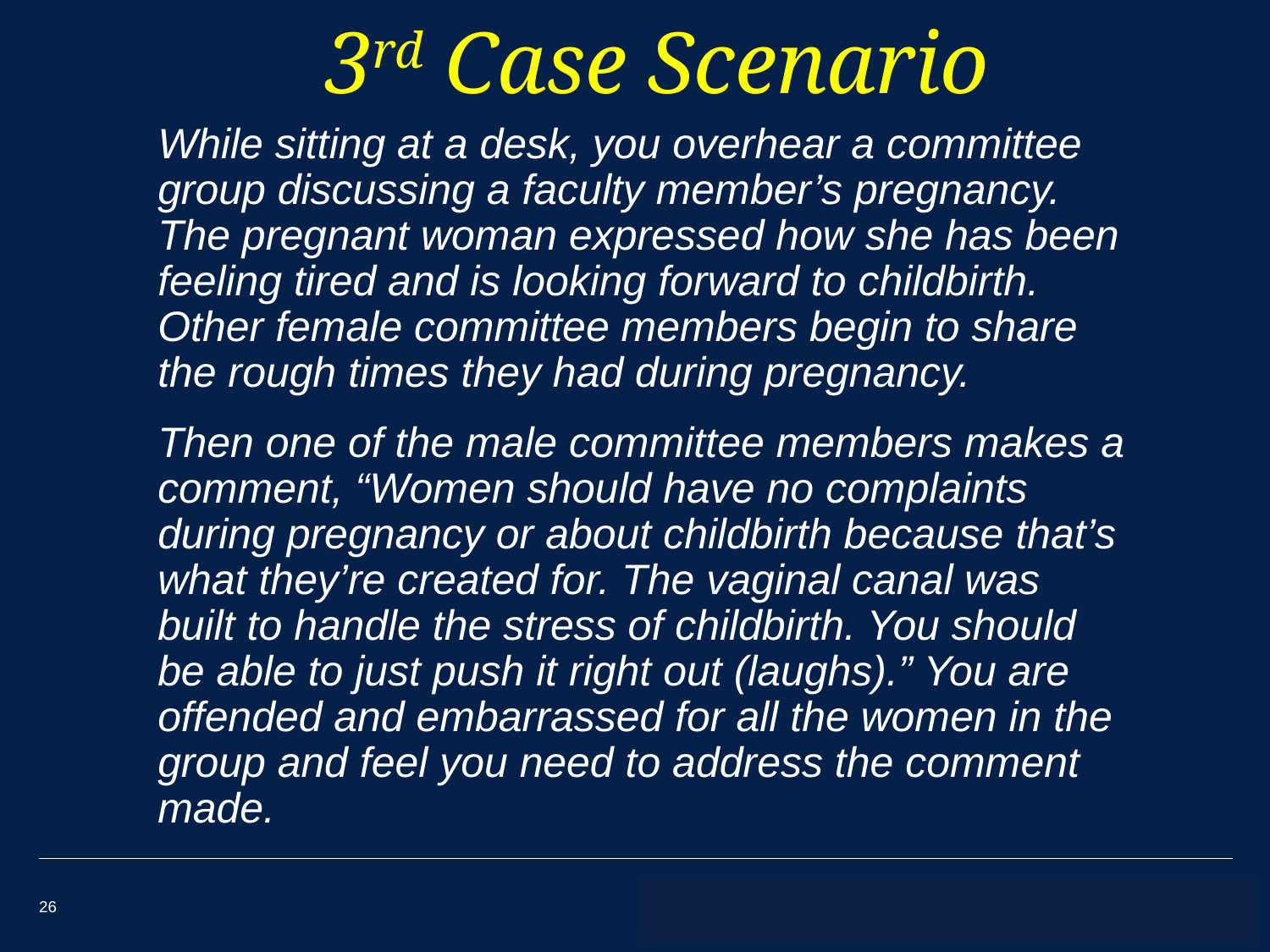

# 3rd Case Scenario
While sitting at a desk, you overhear a committee group discussing a faculty member’s pregnancy. The pregnant woman expressed how she has been feeling tired and is looking forward to childbirth. Other female committee members begin to share the rough times they had during pregnancy.
Then one of the male committee members makes a comment, “Women should have no complaints during pregnancy or about childbirth because that’s what they’re created for. The vaginal canal was built to handle the stress of childbirth. You should be able to just push it right out (laughs).” You are offended and embarrassed for all the women in the group and feel you need to address the comment made.
25

## Slide 27
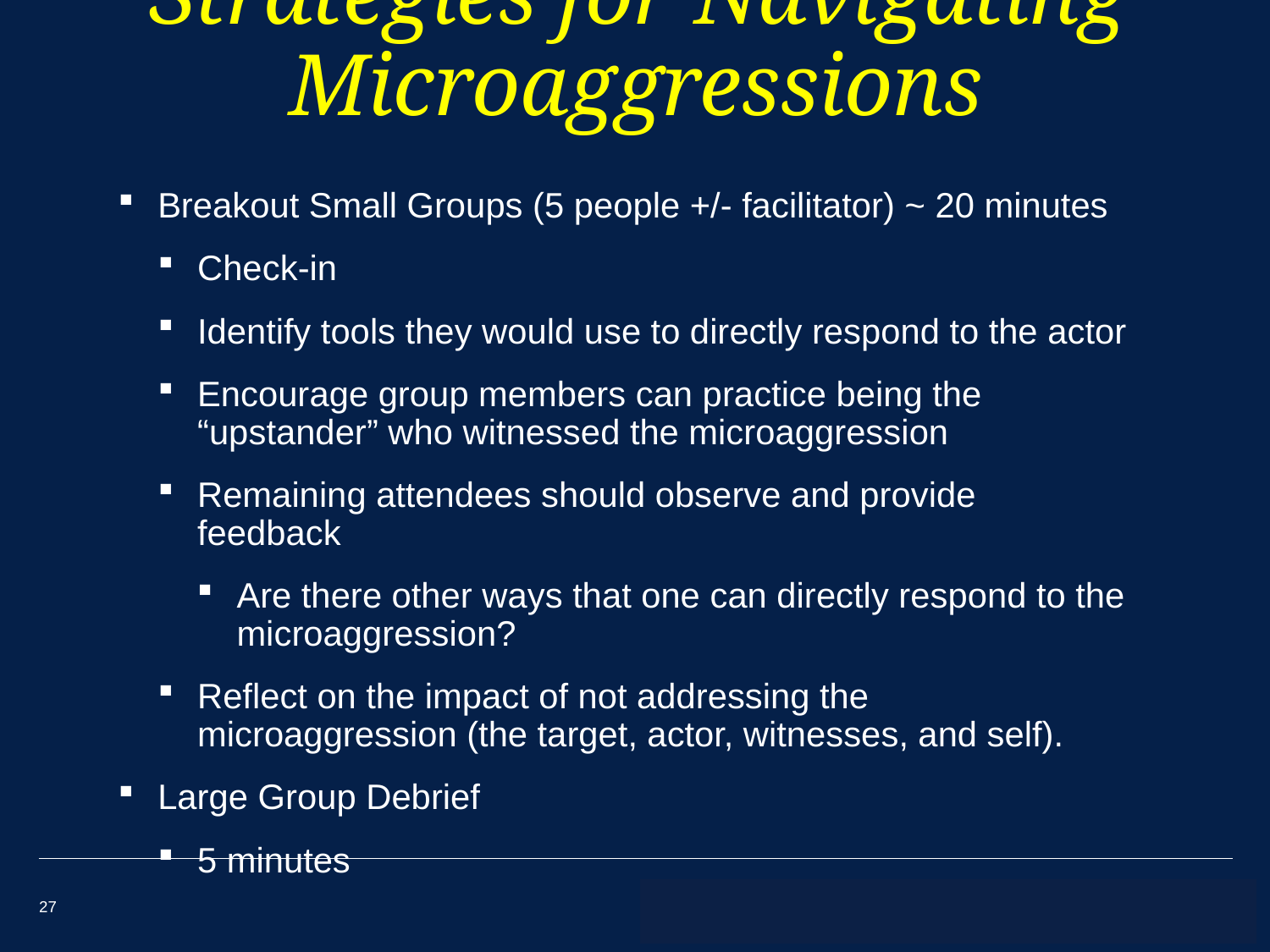

# Strategies for Navigating Microaggressions
Breakout Small Groups (5 people +/- facilitator) ~ 20 minutes
Check-in
Identify tools they would use to directly respond to the actor
Encourage group members can practice being the “upstander” who witnessed the microaggression
Remaining attendees should observe and provide feedback
Are there other ways that one can directly respond to the microaggression?
Reflect on the impact of not addressing the microaggression (the target, actor, witnesses, and self).
Large Group Debrief
5 minutes
26

## Slide 28
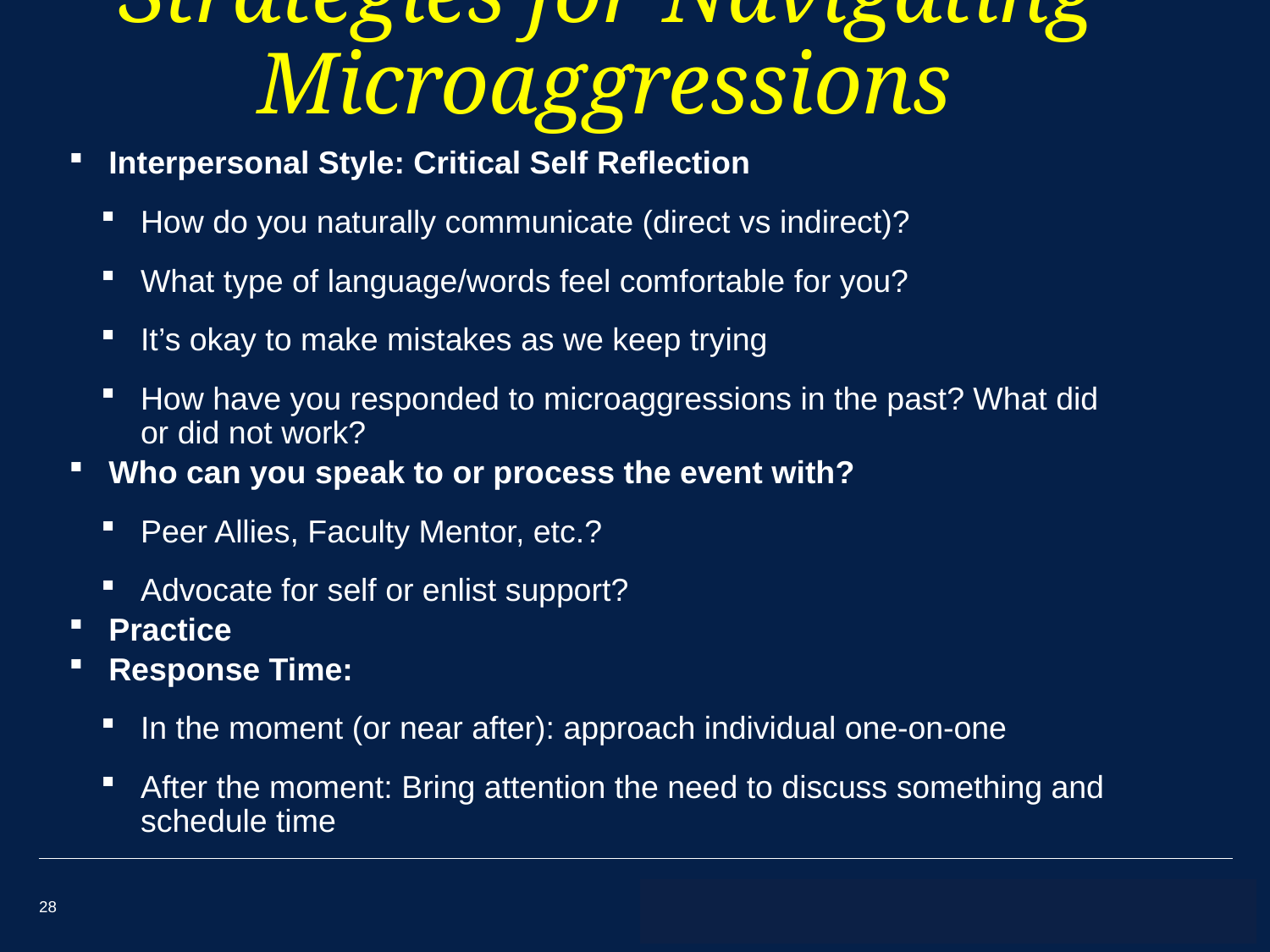

# Strategies for Navigating Microaggressions
Interpersonal Style: Critical Self Reflection
How do you naturally communicate (direct vs indirect)?
What type of language/words feel comfortable for you?
It’s okay to make mistakes as we keep trying
How have you responded to microaggressions in the past? What did or did not work?
Who can you speak to or process the event with?
Peer Allies, Faculty Mentor, etc.?
Advocate for self or enlist support?
Practice
Response Time:
In the moment (or near after): approach individual one-on-one
After the moment: Bring attention the need to discuss something and schedule time
27

## Slide 29
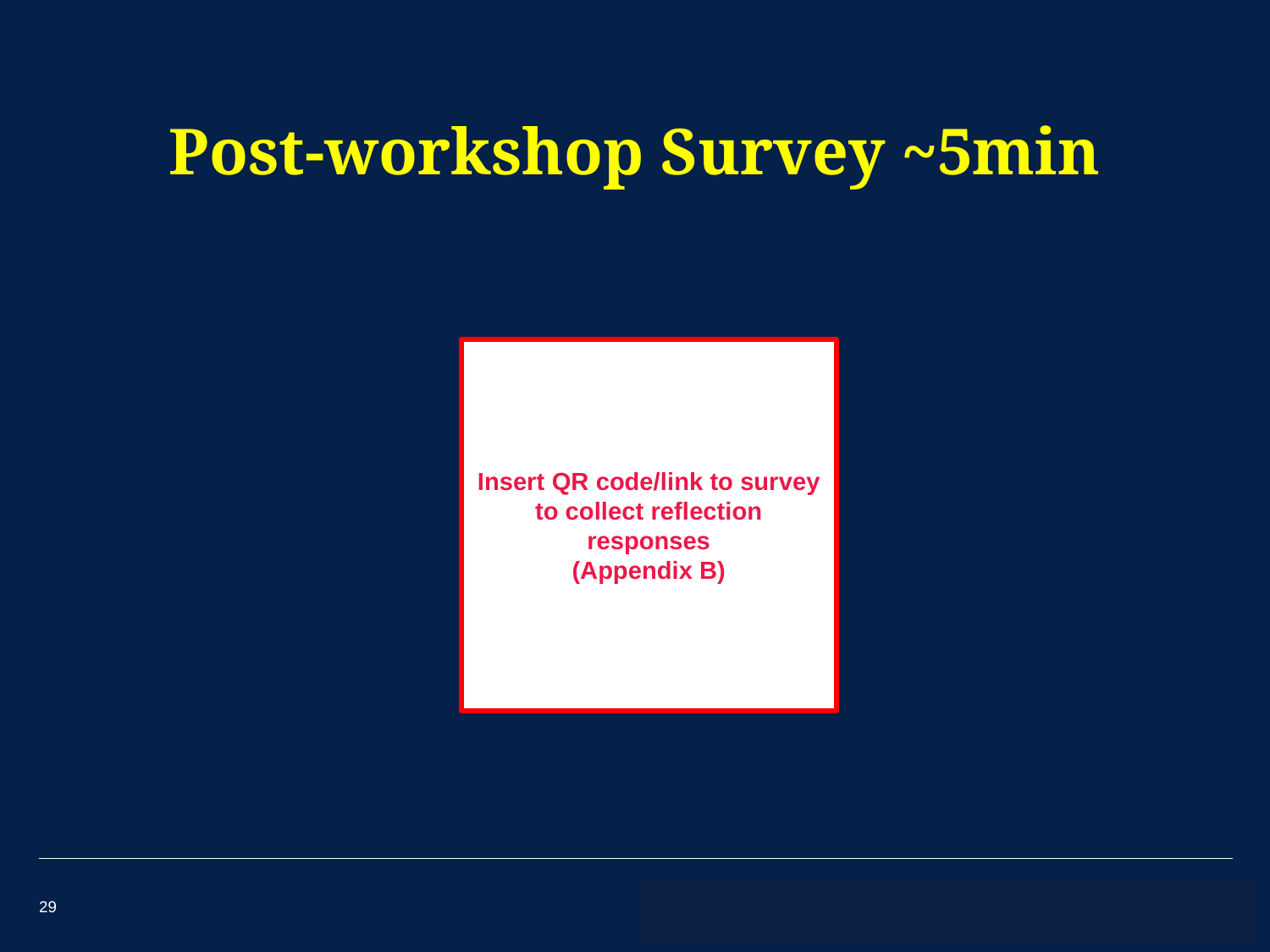

# Post-workshop Survey ~5min
Insert QR code/link to survey to collect reflection responses
(Appendix B)
28

## Slide 30
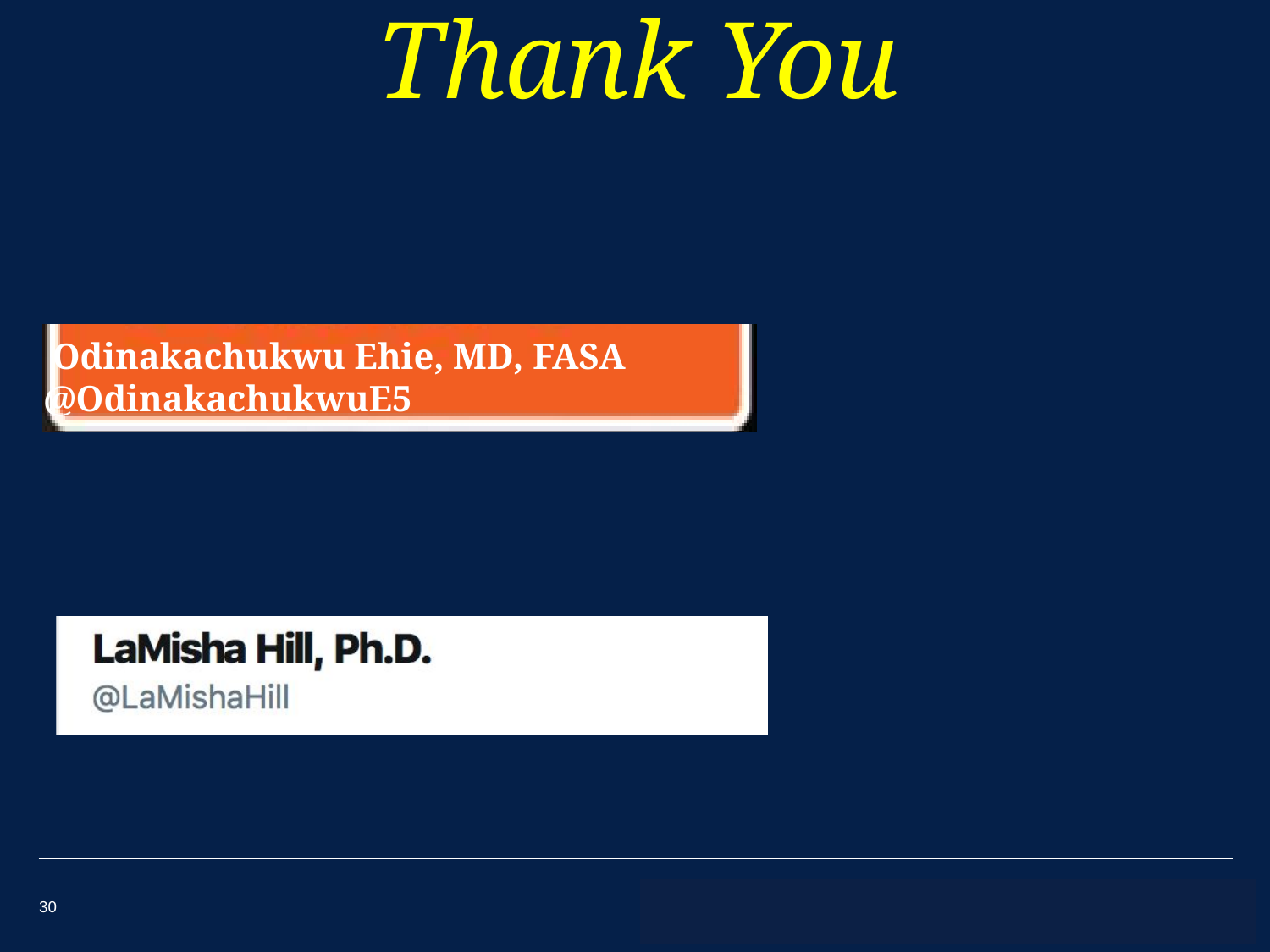

# Thank You
 Odinakachukwu Ehie, MD, FASA
@OdinakachukwuE5
29
