# Supplementary material for: Tools for Addressing Microaggressions: An Interactive Workshop for Perioperative Trainees
Source: MedEdPORTAL. 2023 Nov 28;19:11360. doi: 10.15766/mep_2374-8265.11360 (PMC10682127; doi:10.15766/mep_2374-8265.11360)
Supplement: Supplementary file 1 — Needs Assessment and Presurvey.docxPostsurvey.docxReflective Exercise.docxLearners Guide.docxFacilitator Guide.docxTools to Address Microaggression.pdfMicroaggression Workshop Presentation.pptx [file mep_2374-8265.11360-s001.zip › D. Learners Guide.docx]

**Microaggression Workshop**

Learner’s Guide

Session Dates:

Session Duration: 2 hours

Learners:

Faculty: Small group facilitators

Format: 2-hour group session with small breakout groups

## Session Overview:

In this 2-hour session, learners will be presented current data on microaggression within academic medicine and perform self-reflective small group exercises.

## Curriculum Objectives

By the end of these sessions, learners will be able to:

1. Describe three examples of microaggression in the clinical work environment.
2. Distinguish several strategies for addressing microaggressions.
3. Review case scenarios and apply strategies to address microaggressions and equity themes.
4. Demonstrate one of the five verbal strategies in directly addressing microaggressions within a small group setting.
5. Enhance confidence in managing microaggression as an ally/bystander through role play.

## Pre-Session Preparation

**Required reading prior to workshop:**

- Sotto-Santiago S, et al. “I Didn’t Know What to Say”: Responding To Racism, Discrimination, and Microaggressions with the OWTFD Approach. MedEdPortal 2020; 16:10971.
- Acholonu et al. “Interrupting Microaggressions in Health Care Settings: A Guide to Teaching Medical Students.” MedEdPortal 2020; 16:10969.
- Ehie O, Muse I, Hill L, Bastien A. Professionalism: microaggression in the healthcare setting. Current Opinion Anaesthesiology. 2021; 34(2):131-136.

**Prior to this session facilitators should:**

- Review the Tools for Interrupting Microaggression (Appendix F).
