# Supplementary material for: Tools for Addressing Microaggressions: An Interactive Workshop for Perioperative Trainees
Source: MedEdPORTAL. 2023 Nov 28;19:11360. doi: 10.15766/mep_2374-8265.11360 (PMC10682127; doi:10.15766/mep_2374-8265.11360)
Supplement: Supplementary file 1 — Needs Assessment and Presurvey.docxPostsurvey.docxReflective Exercise.docxLearners Guide.docxFacilitator Guide.docxTools to Address Microaggression.pdfMicroaggression Workshop Presentation.pptx [file mep_2374-8265.11360-s001.zip › E. Facilitator Guide.docx]

**Microaggression Workshop**

Faculty Facilitator’s Guide

Session Dates:

Session Duration: 2 hours

Learners:

Faculty: Small group facilitators

Format: 2-hour group session with small breakout groups

## Session Overview:

In this 2-hour session, learners will be presented current data on microaggression seen within residency programs and perform self-reflective small group exercises.

## Curriculum Objectives

By the end of these sessions, learners will be able to:

1. Describe three examples of microaggression in the clinical work environment.
2. Distinguish several strategies for addressing microaggressions.
3. Review case scenarios and apply strategies to address microaggressions and equity themes.
4. Demonstrate one of the five verbal strategies in directly addressing microaggressions within a small group setting.
5. Enhance confidence in managing microaggression as an ally/bystander through role play.

## Session Outline

| **Duration** | **Topic** | **Who** |
| --- | --- | --- |
| 10 min | Introduction / Goals and Objectives | Facilitator and Learners |
| 10 min | 1^st^ Case Scenario and Poll | Facilitator and Learners |
| 5 min | Large Group Debrief | Facilitator and Learners |
| 15 min | Didactic on Microaggression and Tools to Address Microaggressions | Facilitator and Learners |
| 5 min | Large Group Debrief | Facilitator and Learners |
| 5 min | 2^nd^ Case Scenario | Facilitators and Learners |
| 5 min | Example role play of 2^nd^ scenario | Facilitators and Learners |
| 25 min | Practice tools to directly address microaggression in 2^nd^ scenario within small groups | Small Group of 5 learners + 1 facilitator |
| 5 min | Large group debrief | Facilitator and Learners |
| 3 min | Reflection Exercise | Learners |
| 20 min | Practice tools to directly address microaggression in 3^rd^ scenario within small group | Small Group of 5 learners + 1 facilitator |
| 2 min | Large group debrief | Facilitator and Learners |
| 5 min | Online Post-curriculum Survey | Learners |
| 5 min | Wrap Up: Share ideas for Diversity Curriculum | Facilitator and Leaners |

## Pre-Session Preparation

**Required reading prior to workshop:**

- Sotto-Santiago S, et al. “I Didn’t Know What to Say”: Responding To Racism, Discrimination, and Microaggressions with the OWTFD Approach. MedEdPortal 2020; 16:10971. https://doi.org/10.15766/mep_2374-8265.10971.
- Acholonu et al. “Interrupting Microaggressions in Health Care Settings: A Guide to Teaching Medical Students.” MedEdPortal 2020; 16:10969.
- Ehie O, Muse I, Hill L, Bastien A. Professionalism: microaggression in the healthcare setting. Current Opinion Anaesthesiology. 2021; 34(2):131-136.

**Prior to this session facilitators should:**

- Review the Tools for Interrupting Microaggression (Appendix F).

## Microaggression Session

- Intro in a large group setting **(10 min)**
  - We welcome any thoughts that anyone would like to share before we proceed.
- Review goals and objectives and house rules as a large group
- First case scenario and large group poll ~ consider using interactive zoom poll **(10 min)**
  - - *While doing a pre-op on a male patient for the day of the patient’s surgery. A female nurse anesthetist introduced herself to the patient and began to ask medical questions for their anesthesia planning and discuss the anesthetic plan.*
    - *After introductions to the patient and his support person, the patient initially made superficial misogynist comments. When attempts were made to bring the patient back to the pre-op interview, he looked at his person and suggested he* ***was getting “the bull” angry****, he was going* ***“to take the bull by the horns,”*** *and would laugh as it were merely a joke. The patient continued to make comments referring to his perceived sexual orientation of the CRNA and his concern for being cared for by a “bull”, and then acting as if it were a joke.*
    - *After multiple attempts to keep the course, and continue with the pre-op, the CRNA left the bedside and met with operative team. The attending anesthesiologist spoke with all members of the team and the assignment was modified.*
    - *The* ***surgeon reported that this patient had made multiple comments previously*** *in clinic to a medical assistant who was also part of the LGBT community, but nothing was done in the clinic.*
  - Large group debrief **(5 min)**
- Didactic on Microaggression and Tools to Address Microaggressions **(15 min)**
  - Large group debrief **(5 min)**
- Second case scenario **(5 min)**
  - - *One week after the protests started following George Floyd’s death, a critical care anesthesiology fellow who is a black male arrives at the beginning of his shift to the cardiac intensive care unit. He goes to the room of a patient to introduce himself, and the middle-aged white male patient says, “I am surprised you are here, shouldn’t you be out there rioting with your people?” He responds by saying, “I’m sorry, what did you say?” The patient then responds by saying, “Shouldn’t you be out there rioting with your people?”*
    - *He then proceeds to excuse himself and leaves the room to find a private space.*
    - *A few bystanders heard the interaction, but nothing was said.*
  - Example role play of second case scenario in large group setting **(5 min)**
- Role play of second case scenario in a small group setting **(25 min)**
  - Facilitators to share their screen Appendix F within the small group (Tools: Interrupting Microaggressions)
  - In small group:
    - Facilitators can express that every individual has unique lived experiences and that the responses from these individuals can be quite variable. Encourage learners to incorporate the concepts of power and privilege from the Allyship workshop in October and the importance of “true allyship” as practice being “upstanders” when we are uncomfortable.
    - For individuals that may feel triggered from this case scenario, we want to give you the space to opt out and turn off your camera.
    - Encourage learners to share their name, pronoun, and specialty
    - Check-in about the 2^nd^ case scenario shared in the large group with the learners
    - Identify tools they would use to directly respond to the actor
    - Encourage group members to practice being the “upstander” who witnessed the microaggression
    - Other learners should observe and provide feedback to the variable responses
      - Are there other ways that one can directly respond to the microaggression?
    - Reflect on the impact of not addressing the microaggression (the target, actor, witnesses, and self)
    - Any other questions or comments?
  - Large group debrief **(5 min)**
- Reflection exercise for learners **(3 min)**
  - What tools did you feel most comfortable using in addressing the microaggression in the second case scenario after practicing in your small group setting?
  - [insert link to survey here]
- Role play of third case scenario in a small group setting **(20 min)**
  - - *While sitting at a desk, you overhear a committee group discussing a faculty member’s pregnancy. The pregnant woman expressed how she has been feeling tired and is looking forward to childbirth. Other female committee members begin to share the rough times they had during pregnancy.*
    - *Then one of the male committee members makes a comment, “Women should have no complaints during pregnancy or about childbirth because that’s what they’re created for. The vaginal canal was built to handle the stress of childbirth. You should be able to just push it right out (laughs).” You are offended and embarrassed for all the women in the group and feel you need to address the comment made.*
  - Facilitators to share their screen with the pdf file within the small group (Tools: Interrupting Microaggressions)
  - In small group:
    - Check-in about the 3^rd^ case scenario shared in the large group with the learners
    - Identify tools they would use to directly respond to the actor
    - Encourage group members to practice being the “upstander” who witnessed the microaggression
    - Other learners should observe and provide feedback to the variable responses
      - Are there other ways that one can directly respond to the microaggression?
    - Reflect on the impact of not addressing the microaggression (the target, actor, witnesses, and self)
    - Any other questions or comments?
  - Large Group Debrief **(2 min)**
- Post-curriculum Survey **(5 min)**
  - (consider inserting link to online survey here so facilitators can help distribute)
- Wrap Up **(5 min)**
  - Have residents share ideas for what they would like to see addressed in the diversity curriculum going forward.
  - Name one thing you are taking away with you.
  - (Insert link for word cloud here so participants can see themes that are popular within group)
