# Supplementary material for: Tools for Addressing Microaggressions: An Interactive Workshop for Perioperative Trainees
Source: MedEdPORTAL. 2023 Nov 28;19:11360. doi: 10.15766/mep_2374-8265.11360 (PMC10682127; doi:10.15766/mep_2374-8265.11360)
Supplement: Supplementary file 1 — Needs Assessment and Presurvey.docxPostsurvey.docxReflective Exercise.docxLearners Guide.docxFacilitator Guide.docxTools to Address Microaggression.pdfMicroaggression Workshop Presentation.pptx [file mep_2374-8265.11360-s001.zip › B. Postsurvey.docx]

DEI Microaggressions Post-Survey

Start of Block: Default Question Block

1 Create and enter an anonymous ID: 
1. First 3 letters of your current address
2. Last 2 letters of birth city
3. Last digit of year of birth
4. Number of siblings (not including self)
Example: Red, San Francisco, 1991, 3 brothers = REDCO13

________________________________________________________________

2 The following statements assess your satisfaction towards the microaggressions workshop. How strongly do you agree with the following?

|  | Strongly disagree (1) | Somewhat disagree (2) | Neutral (3) | Somewhat agree (4) | Strongly agree (5) |
| --- | --- | --- | --- | --- | --- |
| This microaggression workshop is important to my training. (1) |  |  |  |  |  |
| I believe this microaggression workshop is relevant to my workplace. (2) |  |  |  |  |  |
| I would recommend this microaggression workshop to my peers. (3) |  |  |  |  |  |
| This workshop has given me *new tools* to address any microaggressions I may encounter. (5) |  |  |  |  |  |

3 The following statements assess your satisfaction towards the microaggressions workshop facilitators. How strongly do you agree with the following?

|  | Strongly disagree (13) | Somewhat agree (14) | Neither agree nor disagree (15) | Somewhat agree (16) | Strongly agree (17) |
| --- | --- | --- | --- | --- | --- |
| The facilitators were well prepared. (1) |  |  |  |  |  |
| The facilitators created a welcoming and inclusive environment for discussions. (4) |  |  |  |  |  |
| The facilitators effectively communicated this information. (5) |  |  |  |  |  |

4 What did you like the most about this microaggressions workshop?

________________________________________________________________

5 What could be improved in this microaggressions workshop?

________________________________________________________________

6 Anything else you'd like to share about your experience in this microaggressions workshop?

________________________________________________________________

7 The following questions are designed to explore your personal experiences related to microaggressions in the workplace.

8 Have you ever **experienced** a microaggression?

- Yes (1)
- No (2)
- I don't know or I'm not sure what a microaggression is. (3)

Display This Question:

If Have you ever experienced a microaggression? = Yes

9 The following questions are designed to explore your experiences **receiving** microaggressions (e.g., between colleagues, patients, etc.) in the workplace, and NOT witnessing microaggressions.

Display This Question:

If Have you ever experienced a microaggression? = Yes

10 Where have you **experienced** microaggressions?

- At your local institution (1)
- Outside of your local institution (2)
- Both (3)

Display This Question:

If Have you ever experienced a microaggression? = Yes

11 In the past 30 days, how often have you **experienced** microaggressions at work?

- 0 (1)
- 1-2 (2)
- 3-4 (3)
- 5-6 (4)
- 7+ (5)

Display This Question:

If Have you ever experienced a microaggression? = Yes

12 What aspects of your identity do these microaggressions involve?

- Race/ethnicity (1)
- Gender (2)
- Sexual Orientation (3)
- Other: (4) ________________________________________________

13 How strongly do you agree with the following?

|  | Strongly Disagree (1) | Somewhat Disagree (2) | Neutral (3) | Somewhat Agree (4) | Strongly Agree (5) |
| --- | --- | --- | --- | --- | --- |
| I know how to define the term *microaggression*. (1) |  |  |  |  |  |
| I feel that I have the tools to address the *microaggression* at the time I receive it. (2) |  |  |  |  |  |
| I am likely to take action at the time I receive a *microaggression*. (3) |  |  |  |  |  |

14 Have you ever **witnessed** a microaggression?

- Yes (1)
- No (2)
- I don't know or I'm not sure what a microaggression is. (3)

Display This Question:

If Have you ever witnessed a microaggression? = Yes

15 The following questions are designed to explore your experiences **witnessing** microaggressions (e.g., between colleagues, patients, etc.) in the workplace, and NOT receiving microaggressions.

Display This Question:

If Have you ever witnessed a microaggression? = Yes

16 Where have you **witnessed** microaggressions?

- At your local institution (1)
- Outside of your local institution (2)
- Both (3)

Display This Question:

If Have you ever witnessed a microaggression? = Yes

17 In the past 30 days, how often have you **witnessed** microaggressions at work?

- 0 (1)
- 1-2 (2)
- 3-4 (3)
- 5-6 (4)
- 7+ (5)

18 How strongly do you agree with the following?

|  | Strongly Disagree (1) | Somewhat Disagree (2) | Neutral (3) | Somewhat Agree (4) | Strongly Agree (5) |
| --- | --- | --- | --- | --- | --- |
| I feel that I have the tools to address the *microaggressions* I witness. (1) |  |  |  |  |  |
| I am likely to take action at the time I witness a *microaggression* addressed towards others. (2) |  |  |  |  |  |
| *Before* this workshop, I felt comfortable confronting microaggressions. (3) |  |  |  |  |  |
| *After* this workshop, I feel comfortable confronting microaggressions. (4) |  |  |  |  |  |

19 The following True/False question is meant to evaluate your general understanding of the terms, and not what you would do personally.

20 Microaggression is the brief subtle snub of any individual that is usually intentional and has negative impact.

- True (1)
- False (2)
- I'm not sure (3)

21 My training up to this point, including this workshop, has prepared me to address conflicts regarding:

|  | Strongly disagree (1) | Somewhat disagree (2) | Neutral (3) | Somewhat agree (4) | Strongly agree (5) |
| --- | --- | --- | --- | --- | --- |
| Race/ethnicity (1) |  |  |  |  |  |
| Gender (2) |  |  |  |  |  |
| Sexual orientation (3) |  |  |  |  |  |

22 In the past year, how frequently did you *confront* microaggressions expressed by patients regarding:

|  | Never (1) | A few times a year (2) | A few times a month (3) | A few times a week (4) | Every day (5) |
| --- | --- | --- | --- | --- | --- |
| Race/ethnicity (1) |  |  |  |  |  |
| Gender (2) |  |  |  |  |  |
| Sexual orientation (3) |  |  |  |  |  |

23 In the past year, how frequently did you *confront* microaggressions expressed by residents regarding:

|  | Never (1) | A few times a year (2) | A few times a month (3) | A few times a week (4) | Every day (5) |
| --- | --- | --- | --- | --- | --- |
| Race/ethnicity (1) |  |  |  |  |  |
| Gender (2) |  |  |  |  |  |
| Sexual orientation (3) |  |  |  |  |  |

24 In the past year, how frequently did you *confront* microaggressions expressed by attendings regarding:

|  | Never (1) | A few times a year (2) | A few times a month (3) | A few times a week (4) | Every day (5) |
| --- | --- | --- | --- | --- | --- |
| Race/ethnicity (1) |  |  |  |  |  |
| Gender (2) |  |  |  |  |  |
| Sexual orientation (3) |  |  |  |  |  |

25 Please answer the following 4 questions in the context of life in general, and not just in work settings.

26 Choose the response that is most applicable to you up to this point:

- I **talk** to others who look like me. (1)
- I **listen** to others who look differently than me. (2)
- I **socialize** with others who look differently than me. (3)

27 Choose the response that is most applicable to you up to this point:

- I **talk** to others who think like me. (1)
- I **listen** to others who think differently than me. (2)
- I **socialize** with others who think differently than me. (3)

28 Choose the response that is most applicable to you up to this point:

- I strive to be comfortable and don't usually address my own biases. (1)
- I understand my own biases and knowledge gaps and share them with others. (2)
- I don't let mistakes from my own biases deter me from continuing to critically evaluate my own biases. (3)

29 Choose the response that is most applicable to you at this point:

- I **avoid** hard questions about privilege and racism. (1)
- I **understand** my own privilege in ignoring racism. (2)
- I **speak out** when I see racism in action. (3)

End of Block: Default Question Block

Start of Block: Demographics

30 Options for identities are abbreviated to preserve survey-takers' anonymity.

31 What department are you in?

- Anesthesia (1)
- Surgery (2)
- Prefer Not to Answer (3)

Display This Question:

If What department are you in? = Anesthesia

32 Which year of anesthesia postgraduate training are you?

- Intern (1)
- CA-1 (2)
- CA-2 (3)
- CA-3 (4)
- Fellow (5)
- Prefer not to answer (6)

Display This Question:

If What department are you in? = Surgery

33 Which year of surgery postgraduate training are you in?

- PGY-1 (1)
- PGY-2 (2)
- PGY-3 (3)
- Research Fellow (4)
- PGY-4 (5)
- PGY-5 (6)
- Prefer Not to Answer (7)

34 Race/ethnicity: (Choose all that apply)

- White/Caucasian (1)
- Black/African American (2)
- American Indian and/or Alaska Native (3)
- Asian (4)
- Native Hawaiian and/or other Pacific Islander (5)
- Latinx (6)
- Other (7) ________________________________________________
- Prefer not to answer (8)

35 Do you identify as Underrepresented In Medicine as defined by your School of Medicine? 

 *Includes: African American/Black, Asian (Filipino, Hmong, or Vietnamese only), Hispanic/Latinx, Native American/Alaskan Native, Native Hawaiian/Other Pacific Islander, or two or more races (when one or more are from the preceding racial and ethnic categories in this list)*

- Yes (1)
- No (2)
- Prefer not to answer (3)

36 Gender Identity: Choose all that apply.

- Male (1)
- Female (2)
- Non-binary (3)
- Transgender (4)
- Prefer to Self-Describe (5) ________________________________________________
- Prefer Not to Answer (6)

37 Do you identify as a member of the LGBTQIA+ Community?

- Yes (1)
- No (2)
- Prefer not to answer (3)

End of Block: Demographics
